# Cryo-Electron Tomography and Proteomics studies of centrosomes from differentiated quiescent thymocytes

**DOI:** 10.1038/s41598-019-43338-9

**Published:** 2019-05-10

**Authors:** Johan Busselez, Francisco Javier Chichón, Maria Josefa Rodríguez, Adan Alpízar, Séverine Isabelle Gharbi, Mònica Franch, Roberto Melero, Alberto Paradela, José L. Carrascosa, José-Maria Carazo

**Affiliations:** 1 Centro Nacional de Biotecnologia (CNB-CSIC), Darwin 3, Campus de Cantoblanco 28049, Madrid, Spain; 2 Present Address: Institut de Génétique et de Biologie Moléculaire et Cellulaire, 1 Rue Laurent Fries, 67400 Illkirch-Graffenstaden, France

**Keywords:** Cryoelectron tomography, Cell division, Proteome informatics

## Abstract

We have used cryo Electron Tomography, proteomics and immunolabeling to study centrosomes isolated from the young lamb thymus, an efficient source of quiescent differentiated cells. We compared the proteome of thymocyte centrosomes to data published for KE37 cells, focusing on proteins associated with centriole disengagement and centrosome separation. The data obtained enhances our understanding of the protein system joining the centrioles, a system comprised of a branched network of fibers linked to an apparently amorphous density that was partially characterized here. A number of proteins were localized to the amorphous density by immunolabeling (C-NAP1, cohesin SMC1, condensin SMC4 and NCAPD2), yet not DNA. In conjuction, these data not only extend our understanding of centrosomes but they will help refine the model that focus on the protein system associated with the centriolar junction.

## Introduction

The centrosome is the main microtubule-organizing center (MTOC) in higher animals^1^, serving as a pole for the mitotic spindle and a hub to regulate mitosis^2^. The centrosome is also a nucleation point for the cytoskeleton and it participates in the attachment of the cytoskeleton to the nucleus^3^. In addition this structure is involved in cell motility^4–6^ and in the spatial organization of quiescent cells^7^. In most quiescent somatic cells, the centrosome nucleates the primary cilium, an important sensor that influences the development of higher organisms^8–10^. Moreover, centrosome dysfunction has been implicated in severe pathologies like cancers^11–13^ and autosomal primary recessive microcephaly, brain dwarfism representing a hallmark of the latter^14,15^.

Metazoan centrosomes are organized around their centrioles, barrel-shaped units constituted by a nine-fold repeat of a microtubule (MT)-based subunit. A typical centriole is ~250 nm in diameter and ~450 nm long^16^. The centrioles are bound together by a network of proteins, the degree of structure and organization of which is poorly understood^17^. These elements are surrounded by a semi-dense proteinaceous cloud called the pericentriolar material (PCM), made up of layers that vary in their protein composition^18–20^. The PCM is a node for different regulatory processes and for the nucleation of cytoskeletal MTs^21^, including the mitotic spindle.

In quiescent cells, the centrosome has two centrioles that differ in age and maturity^22^. The older, more mature of the two centrioles is referred to as the mother centriole, which carries distal and sub-distal appendages^1^. In ciliated quiescent cells, distal appendages serve to attach the centrosome/basal body to the cell membrane^23^. In cycling cells, the centriole begins its duplication during the transition from the G1 to S phase by growing a procentriole orthogonal to its wall^24^. Commencing with the autoassembly of a transient SAS6 structure, shaped as a 9-fold symmetric cartwheel^25,26^, the procentriole then elongates^2^ and each centrosome, formed by a mature centriole and a new immature centriole, subsequently migrates to the poles of the mitotic spindle^17^. Finally, during the maturation phase, immature centrioles become competent for future duplication and the now mature daughter centriole becomes a mother centrioles^27^.

The whole process of duplication requires two types of linkages between the centrioles^28–35^: one between two mature centrioles that is loose and extends over a distance, the association established in quiescent cells; and the other between a mature and an immature centriole that is tight and rigid, maintaining the orthogonal organization inherited from the centriole/procentriole pair. This latter orthogonal organization is relaxed during centriolar disengagement, temporarily blocking the mature centriole duplication site to prevent potentially harmful immediate reduplication^30,31^. During centrosome separation, the link between the mature centrioles is cleaved allowing the two centrosomes to migrate and form separate mitotic spindle poles^15,36^.

The protein system involved in centrosome separation involves different elements. The C-NAP1 protein offers a point of fixation to which rootletin fibers are attached^29^, and the core centriolar protein CEP135 could represent a platform for C-NAP1^37,38^. The fiber-forming proteins LRRC45^39^ and CEP68^40–42^ are also thought to contribute to this system, attaching either directly to C-NAP1 (LRRC45) or through a centlein interface (CEP68)^41^.

Centriolar disengagement involves separase, a cleavage enzyme that is also implicated in sister chromatid disjunction^30^. In humans, separase is known to target the same cohesin substrate in these two apparently related processes^32,43^, yet in other species it remains unclear if cohesin is the only separase substrate at the centrosome^44,45^. Pericentrin^35^ and CEP215/CDK5RAP2^40,46^ may be involved in disengagement through either alternative or complementarily mechanisms^42^. Indeed, despite the use of the same cleavage enzyme, and potentially of the same substrate, other events can desynchronize centriole disengagement and chromatid disjunction^17,33,43^.

Cryo-electron tomography (cryo-ET) of isolated organelles has allowed the centriolar wall of centrosomes from cultured cell to be studied^47^ and by applying sub-tomogram averaging, fine structural details on basal body have been observed in distant species^48–51^.

Given the capabilities of this technique and the benefits obtained from the use of relatively thin specimens, we have used cryo-ET to analyze mammalian centrosomal ultrastructure in a hydrated state. Due to their minimal PCM and the release of centrioles from the orthogonal position, centrosomes purified from young lamb thymus^52,53^ are well suited to such analysis. Moreover, as the thymus contains mostly of quiescent, differentiated thymocytes, the population of centrosomes extracted is homogeneously mature. In addition, the centrioles from thymocyte do not form cilia. This study also takes advantages of a new generation of electron detectors^54^, and more advanced denoising approaches^55^. These technical advances facilitate the characterization of poorly organized features^56^ that are not amenable to symmetrization or averaging, permitting the analysis of the individual centrosome. Accordingly, we adopted an approach that is particularly suited to study inter-centriolar linkage. To complement these structural studies,we carried out proteomic studies on the enriched centrosomal fractions from young lamb thymocytes, using mass spectrometry(MS) to evaluate the complete proteome of these centrosomes. Finally, the data obtained were compared with existing information extracted from cultured KE 37 cell centrosomes, obtained from less differentiated, cycling cells^57–59^.

## Results

Lamb thymus centrosomes were isolated on sucrose gradients and fractions from the second gradient (see Methods) were studied by immunofluorescence, proteomics and cryo-ET. We used a MS-based proteomic analysis to determine the protein composition of the centrosome from quiescent and differentiated thymocytes. In addition, immunolabeling clarified the localization of a number of proteins.

### General structural characterization

**Figure 1:**
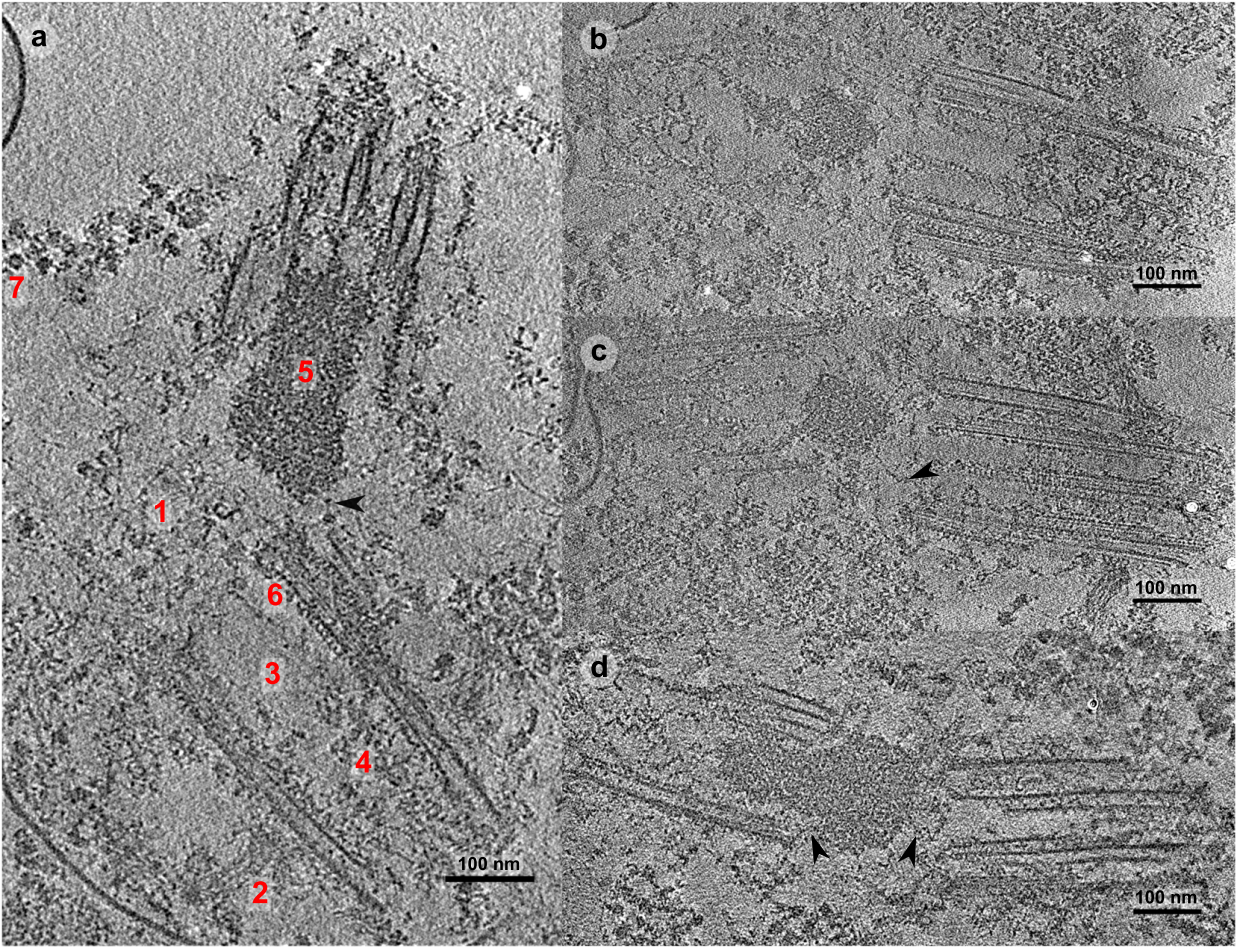
(**a)** Typical slide of a cryotomogram from isolated lamb thymus centrosomes. The two centrioles are disengaged and linked by a loose, branched network of fine fibers (1). The sub-distal appendages (2) distinguish the mother centriole and the proximal lumen (3) appears empty, whereas the distal lumen contains some structures (4). An amorphous density (5) is shown between the two centrioles, the precise localization of which is variable in the isolated centrosome reconstructions. A regular pattern (6) is visible along the centriole wall with a repeat distance measured as ~83 Å. Some denser assemblies are visible (7) in the very weak pericentriolar material (PCM), probably protein complexes. (**b**–**d)** Slides of other tomograms are shown in which similar features are observed. The arrowheads point to some fibers that are located between the two centrioles and that appear to be linked to the amorphous density.

We reconstructed and analyzed 17 high quality CTF (contrast transfer function) -corrected cryo-electron tomograms of lamb thymus centrosomes, that showed representative structural features (Fig. 1a). While there was virtually no semi-amorphous form of PCM^18–20^ surrounding the two centrioles, some protein complexes could be hinted within the very weak PCM (red 7). The two centrioles appeared to be connected by a fibrous network (Fig. 1a red 1, Suppl. Video 1) and sub-distal appendages (red 2) distinguished the mother centriole, which had a relatively empty lumen in the proximal half (red 3) and a more electron dense lumen in the distal half (red 4). An apparently amorphous density (red 5) was observed between the two centrioles in almost every cryo-tomogram, consistent with earlier indications^52^. This material was apparently linked to the centrioles by some fibers (Fig. 1 arrowheads), although it was positioned distinctly in the different reconstructions (Fig. 1), and was not observed in a pair of cryo-tomograms (Suppl. Video 1).

As indicated elsewhere, there is a variable amount of flattening at the level of the centrioles in the cryo-tomogram of isolated centrosomes or basal bodies^47–51^ and indeed, there was a degree of flattening evident in most of our tomograms (Suppl Fig. 11). However, in the majority of the affected tomograms the centriole flattening ranged from little to 30%, and up to 30 and even 40% in a few.

### Proteomics characterisation of lamb thymus centrosome

Among the 40, 50 and 70% sucrose fractions, the latter had most centrosome but also, more contaminants (Suppl. Fig. 1a). As such, the best compromise for analysis when assessed by electron microscopy(EM) appeared to be the 50% (Suppl. Fig. 1b).

The proteome of all three fractions was characterized by gel-Liquid Chromotography (LC)-MS.

**Figure 2:**
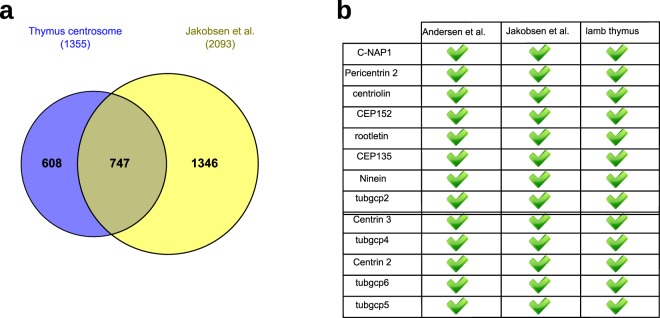
(**a)** Quantitative comparison between the proteins detected by mass spectroscopy (MS) in centrosomes isolated from KE37 cells (right circle)^58^ and the proteins detected by MS in centrosomes isolated from lamb thymocytes (left circle). The total number of proteins in each dataset is shown in parentheses. (**b)** Panel of well-characterized centrosomal proteins. Top, proteins readily detected in published proteomic studies are shown with a high z-score^57^ or a large number of peptides^58^. Bottom, proteins with low scores in the same studies (following the same criteria).

The initial analysis (Suppl. Table 1) allowed the list of proteins identified to be compared with other proteomics studies, such as that in which a centrosome preparation from KE37 lymphoblasts was analyzed^58^ (Fig. 2a). Initially, we noted that fewer proteins were detected here than in the KE37 centrosomes^58^. It is generally considered that centrosome isolation by sucrose gradient enriches rather than purifies, and that the samples obtained still contain many contaminants. Given the weak cytoskeleton of the thymocytes compared to KE37 cells^60^, the thymus system would seems to be well suited for centrosome enrichment. In addition, our protocol to prepare centrosomes involved two sucrose gradient steps rather than the single step used elsewhere^57,58^, which tends to reduce contaminants, as observed by proteomic in the isolation of Chlamydomonas basal bodies^61^. Indeed, we observed little cell debris when assessed by EM (Suppl. Fig. 1b) and in particular, little chromatin was detected by immunofluorescence (Suppl. Fig. 1a).

We compared the proteome obtained in the 40%, 50% and 70% fractions (Suppl. Fig. 2), considering the proteins at the intersection of the three fractions to be the strongest candidate centrosomal proteins, a group comprised of around 670 proteins.

We established a panel with the centrosomal proteins at the two extremes of the detection ranges used previously^57,58^ (Fig. 2b), using the criterion being the z-score^57^ or the number of peptides^58^. All these proteins were detected in our analysis of the thymus centrosome indicating that the sensitivity of our experimental approach is comparable to that of previous studies.

**Figure 3:**
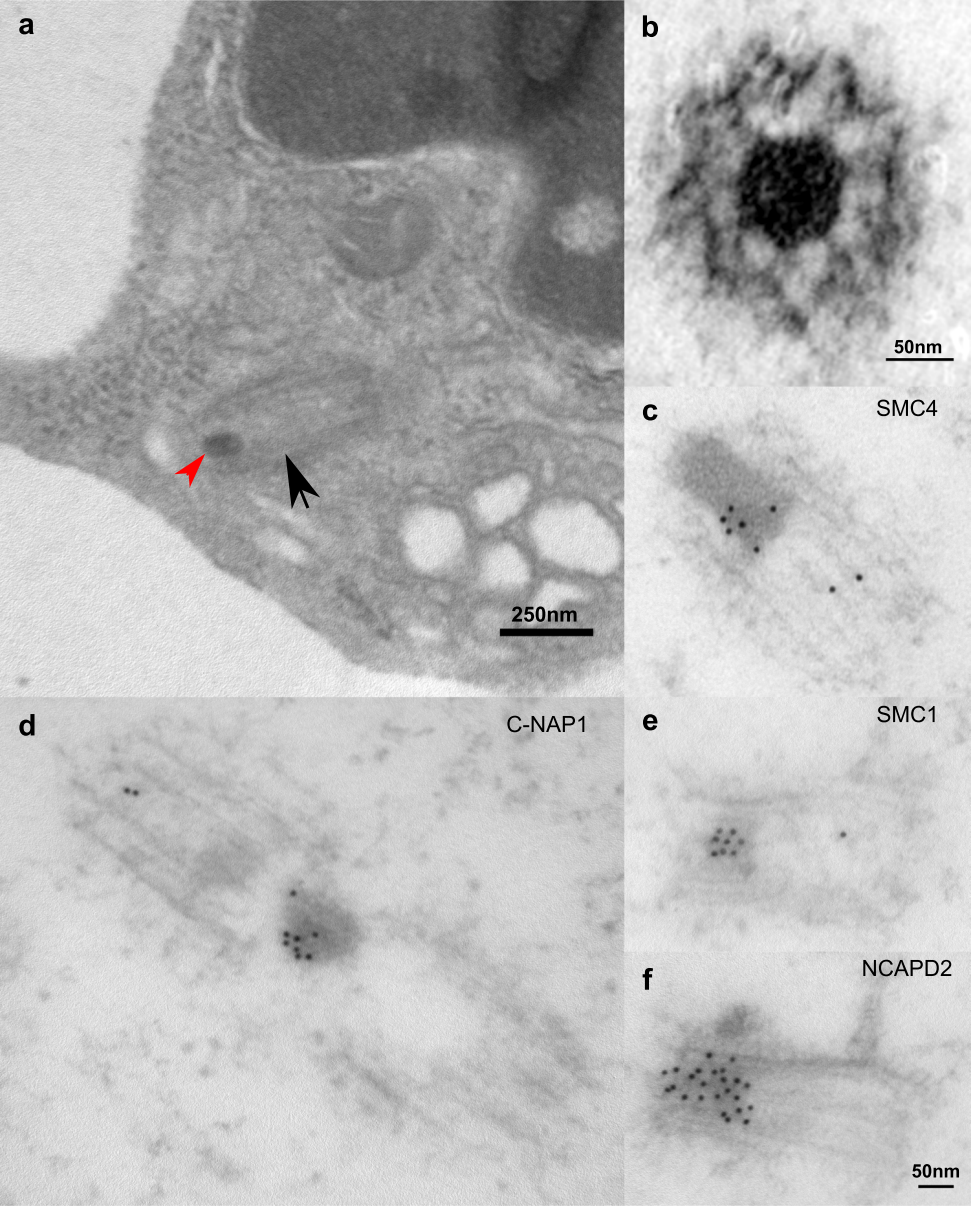
(**a)** Harvested calf thymocytes were fixed chemically, resin-embedded and ultrathin-sections were obtained. Some sections contain centrioles (black arrow) with an amorphous density (red arrowhead) similar to those described in cryo-ET images of isolated thymus centrosomes. (**b**) Resin inclusion of the centrosome pellet sedimented after lysis in which a similar amorphous density is evident. Note that this density is usually present at only one of the centrioles and it appears to be attached to the centriolar wall by linkers. This density is immunolabeled for SMC4 (**c**), C-NAP1 (**d**), SMC1 (**e**) and NCAPD2 (**f**).

In a quantitative shotgun-MS proteomic study of centrosomes from KE37 cells^59^, a set of centrosomal proteins was ranked in terms of copy number per centrosome (see Fig. 3 there in^59^ and Suppl. Fig. 3a. here). A set of 4 low abundance proteins were not detected in our study (PLK4, STIL, CSPP1 and ODF2L), raising the questions as to the limits of sensitivity. However, PLK4 is a kinase that triggers centrosome duplication at the onset of S-phase^62^ and STIL is a transient protein associated with the cartwheel formation^63,64^, which might explains why these two proteins are not detected in centrosomes from quiescent, non-ciliated, thymocytes. A similar case could be made for CSPP1^65,66^, while ODF2L /BCAP appears to be a satellite protein^67^. In this context, it may not be surprising that these 4 poorly abundant proteins are not found at the quiescent thymocyte centrosome. In terms of the remaining spectrum of abundance (Suppl. Fig. 3a), few proteins are missing and those that were dispersed over the whole spectrum, with no further indication our analysis might be compromised in terms of sensitivity.

In terms of the main focus of this study, the inter-centriolar junction, all the linker protein previously listed^59^ were detected in the centrosome from lamb thymocytes (Suppl. Fig. 3b).

We indicated above that the purification protocol used here might identify fewer proteins than those found previously. Moreover, the biological context of differentiated, quiescent thymocyte differs considerably from the proliferating, less-differentiated KE37 cells.

To explore this idea, we examin the differences in protein composition and in their relative function indicated by their associated gene ontology terms. The lists of proteins identified in lamb thymus or KE37^58^ centrosomes were subjected to an enrichment analysis centered on biological processes (Table 1, Suppl. Tables 2, 3), grouping the processes into “lists” of differing degrees of implication, and comparing our protein set to those detected previously in KE37^58^ (Suppl. Tables 2, 3). As an alternative filter for known centrosomal proteins, both the lists of genes reported previously in KE37 cells^58^ and those identified here were compared to the list of genes considered to code for proteins mapped to the centrosome in the human protein atlas^68^.

**Table 1:** Extract from Supplementary Tables 2 and 3.

KE37 cells	Both	Lamb thymus
**Centrosome duplication (BP) GO:0051298**		
SASS6	CETN2(Centrin 2)	KIAA0753
NDE1	CCP110	
PLK4	CEP192	
CENPJ	CEP152	
STIL	C2CD3	
CEP72	TUBGCP3	
	CNTROB (Centrobin)	
	TUBGCP6	
	CDK5RAP2	
	CEP63	
	CEP135	
	TUBGCP2	
	TUBGCP4	
	TUBGCP5	
**Regulation of centrosome cycle (BP) GO:0046605**		
KE37	Both	Lamb thymus
AURKA	SPICE1	CHMP4B
PLK4	CEP76	XPO1
CENPJ	CEP131	VPS4B
	CDK5RAP2	CHMP2A
	CEP120	CHMP1B
	NEK2	CEP85
		KIF11
		NPM1
		RBM14

Comparison between Gene Ontology terms related to the Centrosome duplication (BP) and Regulation of centrosome cycle (BP) pathways (GO:0051298 and GO:0046605, respectively) obtained by gene enrichment comparison of the list of centrosomal proteins in KE37 centrosome sample^58^ and those we detected in the lamb thymus centrosome.

Left column, genes that are only found on the existing list of centrosomal genes^58^. Center column, genes that appeared previously^58^ and in our data. Right column, genes that were only found here.

In terms of the centrosome associated processes, our thymocyte centrosome preparation did not contain CPAP (CENPJ), SAS6 and STIL, while the CEP68 interactor centlein was not observed in either of the two studies.

### The apparently amorphous density inside the centriole

In most of our cryo-tomograms, a density with no obviously identifiable substructure, was clearly evident inside one of the centrioles, although its exact localization within the vicinity of the centrioles varied (Fig. 1). Granular material has previously been reported in this region^52^, although this feature has not generally been contemplated in the scheme of centrosome ultrastructure. We ruled out that this phenomenon might be an artifact generated by the purification protocol by examining ultrathin-sections of chemically fixed and resin-embedded cells harvested from the calf thymus (Fig. 3a). In addition, the pellet from the lamb thymocyte lysate was submitted to mild fixation, plunge freezing and freeze-substitution prior to obtaining ultrathin-section (Fig. 3b). Similar apparently amorphous densities were apparent in the centrosomal pellet and in whole cells, although the effect of the fixation protocol may have made them appear denser and more sharply delimited than in cryo-microscopy. The density was mainly found inside one of the centrioles in pellets and whole cells, whereas it was more widespread in the cryo-tomograms of isolated centrosomes (Fig. 1). Indeed, in sections and in some cryo-tomograms, it also appeared to be attached to the centriole wall by linkers (Fig. 3b, Suppl. Fig. 6, red arrowheads).

To determine the composition of this apparently amorphous feature, we used immunogold EM to study the location of a number of proteins (Fig. 3c–f). C-NAP1/CEP250 and SMC1 were clearly located in the apparently amorphous density and theses proteins also appeared to associate with other structural elements of the centrosome, albeit more weakly. Having identified SMC1 as an element of the apparently amorphous density, we assessed the presence of condensin (Fig. 3c,f). There is considerable evidence that condensin and cohesin related complexes have functions beyond the scope of condensing chromatin and the binding of sister chromatids, respectively^32,43,69,70^. In our proteomic analysis, we detected both the core condensin protein SMC4 and the condensin complex subunit 1 (NCAPD2), a protein that makes part of the condensin I complex. Indeed, immunofluorescence analyses situated both these proteins at the purified centrosome and in immunogold EM analysis, a strong labeling was evident for both within the apparently amorphous density (Fig. 3c,f).

Condensin and cohesin are related protein complexes originally identified by their involvement in DNA-shaping processes. Their presence in this apparently amorphous density raised the question of the possible presence of DNA. Results from immunogold electron microscopy using the anti-DNA antibody clone AC-30–10 were negative, an antibody repeatedly reported to target double- (ds) and single-stranded DNA as well as zDNA^71^. Additional assays using another two anti-dsDNA antibodies were also negative (Suppl Fig. 10).

### The intercentriole system

The small amount of PCM specific to the thymus centrosome allowed us to visualize a network of fibers connecting the two centrioles (Fig. 1a, red 1). This set of fibers appeared to be more extended in the near-native cryo conditions than in images of resin-embedded samples, and some of the fibers were located between the two centrioles apparently linked to the amorphous looking density (Fig. 1, arrowheads). Mild denoising of the cryo-ET reconstructions represented the fiber system as a branched network of straight lines (Suppl. Figs 4–6, Suppl. Video 1). Antibodies to proteins identified in our proteomics study and reported as possible constituents of this network system^29,35,39–41^ were used in an immunofluorescence screening. Rootletin, LRRC45, and CEP215/CDK5RAP2 consistently produced fluorescence signals (Suppl. Fig. 9), although a reliable immunogold signal was not obtained with these antibodies. This discrepancy may reflect the restricted antibody access to antigen for post-embedding labelling, only recognizing those available at the surface of the section. Moreover, beyond the areas with a high antigen density, such as the amorphous looking density, post-embedding labeling may also give a weak signal and indeed, the branched network of fine fibers is particularly likely to have a low concentration of accessible antigen at the surface of the section.

## Discussion

Using non-native techniques like chemical fixation and freeze substitution, a vision of the centriolar junction has been constructed, deriving a structural organization in which rootletin and C-NAP1 form fibers and attachment points, respectively^72^. Other interactors have been identified, such as CEP68 and CEP215/CDK5RAP2^42^ or LRRC45^39^, although the general structural model remains almost unchanged. The lack of high quality structural information gathered in near-native conditions motivated us to study the mammalian thymus centrosome by cryo-ET. An earlier cryo-EM study showed that thymus centrosomes are well suited to visualize the intercentriolar space in cryo conditions^73^, making them a system of choice to study the mechanisms at play at centriolar junctions. Through this approach, we reconstructed high quality cryo-tomograms that showed centrioles apparently bound by a disperse system of fibers, which nested a denser, rounded material with no discernible substructure under our experimental conditions.

A variable degree of flattening has been seen when Cryo-ET of centrioles have been performed^47–51^. Here, we have prepared our cryo grids with an ice layer thick enough to comfortably host the isolated thymus centrosome, while still thin enough to give a suitable signal to noise ratio. However, most of our centriole tomogram are affected by moderate flattening (between a few percent and 30%) (Suppl. Fig. 11) and a few of them present higher flattening (between 30 and 40%). Reducing the surface tension at the air water interface, for example with low concentration of low Critical Micelle Concentration (CMC) detergent in the final sample buffer, could be explored in the future to redude this flattening effect.

The distal part of the lumen is occupied by a protein density (Fig. 1a, Suppl. Figs 8, 9), and it has been proposed that these protein assemblies may have a regular shape, forming a disc stack or helix^74^. Our data indicate a very complex assembly and/or a very irregular structure, even in the tomograms with little flattening of the centrioles (around 10%).

The system that connects the two centrioles appears to be constituted by at least two major parts, one being a system of fibers (Fig. 1, Suppl. Figs 4–6, Suppl. Video 1). Our cryo-ET data show no evidence of a fiber system that directly binds the two centrioles but rather the fibers are dispersed and form a network with the appearance of straight branching of elements (Suppl. Figs 4–6, Suppl. Video 1), that is likely to have rather distinct mechanical characteristics.

This fiber system accommodates a more or less rounded assembly, which appears to be denser and more compact than the PCM observed for proliferating KE37 cell centrosome in cryo-conditions^75^.

In a study of chemically fixed and resin embedding calf thymus centrosome^52^, a nodule in the PCM was described in the following manner: “A globular domain can be seen at the junction between the two centriole”. However this globular domain appears significantly smaller and denser that the apparently amorphous density visible in our cryo tomogram. Indeed this globular domain was seen to present structures that led the authors to suggest that it “could be a folded form of the intercentriolar link”.

As no such apparently amorphous and voluminous density has since been reported between the centrioles, we further tested the lamb thymus centrosome pellet before passing it through the sucrose gradient steps, as well as whole calf thymus cells. The pellet was freeze-substituted and the whole cells were fixed chemically fixed for resin inclusion, before sections were obtained and visualized by EM. Similar densities were found in the centrosomes from pellets and calf thymocytes, and in both cases, the densities appeared amorphous but more compact than in the cryo conditions, probably due to the fixation process used. It is possible that the source of discrepancies between the apparently amorphous density found in our cryo tomograms and the globular domain described previously^52^ reside in the presence or absence of a chemical fixation step in the sample preparation. This discrepancy may also be due to the presence of EDTA in the buffer used previously^52^, which has since been shown to induce an important remodeling of the PCM^16^. Consequently, EDTA was not included in any the buffers used here.

Some connections between the density and the centriolar walls were detected, both in cryo-ET reconstructions and in chemically fixed samples.

In cryo-tomograms from isolated centrosomes, only some of the apparently amorphous densities were detected within a centriole, whereas they were almost always visible in a centriole after chemical fixation of whole cells or the centriole pellet. This discrepancy might reflect the influence of a step in the isolation procedure that improve the dispersion of centrosomes when they are resuspended from the pellet using a needle. However, when performed excessively this step is known to damage the inter-centriolar system and can even tend to favor the harvesting of isolated individual centrioles (Veronique Chevrier, personal communication). Although this step was carried out with care, the binding of the density within the centriole might be fragile and easily disturbed, which could also explain why in occasion the apparently amorphous density was completely stripped away from the centrosome, in our hands. Indeed, this may also explain why the globular domain was previously reported at various sites in the isolated centrosome^52^.

To determine which proteins form part of the fibers and the apparently amorphous system, and to track differences in centrosome composition between quiescent differentiated cells and dividing, less differentiated cells, we performed a proteomic study of the centrosomes isolated from the lamb thymus. In accordance with observations from denaturing gels^52^, fewer proteins were detected than in previous studies on KE37 centrosome^57,58^. The quality of the data was confirmed by the presence of some well-established centrosomal proteins, those readily detected previously^57,58^, and of proteins near the limits of detection in those studies. All the proteins in this panel were detected here, suggesting a comparable sensitivity. When compared with data from a quantitative proteomic study on the KE37 centrosome^59^ there was no significant diference in the limit of sensitivity to the detect centrosomal proteins.

Since centrosome enrichment on sucrose gradients does retain contaminants to some extent, we used a gene ontology approach to compare our centrosome proteomic data with the list of centrosomal proteins reported earlier^58^. Enrichment was compared for the biological processes in which the centrosome is implicated. As anticipated, the SAS6 cartwheel protein was not detected, nor were the centrosome duplication related CPAP and STIL. Moreover, the PLK4 regulator detected previously in KE37 cells^58^ was also absent here.

Based on our proteomic data and that in the literature, we selected a set of candidate proteins and studied their localization by immunogold EM, including: C-NAP1 that is thought to be the main fixation point for fibers between the two centrioles^42^, the fiber candidate rootletin, CEP68 and LRRC45, as well as CEP135 as a possible platform for C-NAP1, and cohesin (SMC1), CEP215 and pericentrin as possible targets of separase.

The C-NAP1 protein and SMC1 localized to the apparently amorphous density, althought their distribution appeared to differ slightly, with C-NAP1 was found closer to the periphery of the density and SMC1 at its center. Condensin, a protein complex related to cohesin and now reinterpreted to be quite versatile, was also identified, as indicated by the detection of two of its component proteins, SMC4 and NCAPD2.

Here we present new structural information about the dynamic protein system that binds the centrioles obtained in a model of quiescent and differentiated cells. This system involves a branched network of fiber that apparently nests an amorphous looking density, which itself appears to be linked to the internal part of the proximal lumen of the centriole by fibers. The precise nature of the fibers constituting the branched network remains to be determined, as does the identity of the protein joining the apparently amorphous density to the centriole. Some elements of the apparently amorphous density have been identified here, showing it to be versatile in the sense that it contains proteins targeted to centriolar disengagement (SMC1 in the center), as well as proteins associated with fixing the centrosomal linker to the centriole (C-NAP1 at the periphery). This structure may be important to further understand the mechanisms associating centriolar disengagement with the constitution of the fiber network^17^ to which the apparently amorphous density also appears to be attached.

Centrosomal separation is a process achieved by a combination of cleavage (Nek2 pathway) and mechanical stress (through the action of Eg5 kinesin). Elucidating the centrosomal linker structure will be necessary to establish a chemo-mechanical model from which the exact contribution of these two pathways, can be determined. This goal will require a further structural characterization in near native state using also other cell systems, gaining more detailed information about the localization of the proteins involved. In addition, it is likely that all this information will have to be unified through in silico modeling.

In summary, we have studied here the composition of the centrosome from lamb thymus, an interesting model of quiescent and differentiated cells, through a MS proteomic analysis. The data obtained were compared with those obtained from a similar analysis in proliferating and less well differentiated KE37 human cells, and ordered in accordance with biological processes delimitation obtained from gene ontology annotations. The information obtained is likely to be helpful to establish a comparative framework of centrosomal proteins in quiescent and cycling cells, and in differentiated (non-ciliated) versus less differentiated cells.

## Material and Methods

### Antibodies

The following antibodies were used in this study: mouse anti-beta-tubulin (T4026) and -NCAPD2 (SAB1404764: both from Sigma); rabbit anti-C-NAP1/CEP250 (14498-1-AP), -SMC4 (24758-AP: both from Proteintech); rabbit anti-SMC1 (A300-055A), -CEP215/CDK5RAP2 (IHC-00063: both from Bethyl); rabbit anti-rootletin/CROCC (SC-135275: Santa Cruz); rabbit anti-rootletin (R145)^29^ and -CEP215/CDK5RAP2 (R174)^40^ antiserum, courtesy of Erich Nigg (Biozentrum, Basel, Switzerland); mouse anti-DNA clone AC-30-10 (CBL186: Millipore); human anti-double-stranded DNA (LS-C64820/22140: LSBIO); and murine anti-double-stranded DNA (MA1-35346: ThermoFisher).

### Centrosome purification

Centrosomes were purified from freshly extracted lamb or calf thymus as described previously^52^. Briefly, thymus tissue was gently mashed in a 0.4 mm sieve, and the collagen was removed by filtration through a 0.2 mm sieve. The cell suspention was centrifuged and filtered through a nylon mesh, and the cells were washed by two-step of centrifugation and nylon mesh filtering, and disrupted in 1.5 or 3 L of hypotonic buffer at a concentration of 10^7^ cells/ml. Aggregates were sedimented and after rectifying the pH of the supernatant, the centrosomes were pelleted by centrifugation. The centrosome-enriched pellet was resuspended, dispersed gently with a needle and loaded on a sucrose gradient (30%, 40%, 50% and 70%). Centrosome-rich fractions were collected, diluted and concentrated on a narrower step sucrose gradient (40%, 50% and 70%). The highest concentration achieved was 2 × 10^9^ centrosomes/ml. EDTA was excluded^16^ from all the buffers and reagent used^52^. In this study 10 independent samples were purified from 7 lambs and 3 calves, each studied separately. For proteomic studies, negative stain and cryo-EM approaches, the full protocol was applied to obtain a solution of isolated centrosomes. For resin inclusion and immunogold microscopy, the centrosome pellet obtained after the lysis was used, recovering this centrosome-enriched pellet carefully with a spatula and transferring it to an Eppendorf tube.

Centrosomes were isolated from tissue samples (thymus) obtained post-mortem from lambs processed at an authorized slaughterhouse (Matadero de San Agustin de Guadalix, Madrid, Spain). According to the current EU and National legislation, the use of these post-mortem animal tissues does not require the authorization of an Ethics committee.

### Light microscopy

Up to ~4 × 10^6^ centrosomes were sedimented on a glass coverslip and fixed with methanol (6 min, −20 °C) or 4% paraformaldehyde (12 min, room temperature). A standard immunolabeling procedure was followed, using Alexa 488 or Alexa 594 conjugated secondary antibodies (ThermoFischer Scientific). DNA was systematically stained with DAPI (Sigma) and the coverslips were mounted with ProLong Gold (ThermoFischer Scientific). Mounted coverslips were examined under a fluorescence microscope Leica DMRXA and visualized at 25 °C on a confocal microscope Leica TCS SP5 with a 100X/1.4 NA immersion oil objective monitored through the Leica LAS AF v2.7 acquisition software. Image processing included a median filtering of each channel (radius = 3) and calculation of the maximum projection over the confocal stack. Contrast was adjusted automatically in ImageJ^76^.

### Ultrastructural studies

For ultrastructural EM studies, cells were isolated from fresh thymus tissue, centrifuged onto coverslips and fixed *in situ* for 1 h at room temperature with a mixture of 2% paraformaldehyde and 2.5% glutaraldehyde (both from TAAB). The cells were then post-fixed with 1% osmium tetroxide in PBS (45 min), treated with 1% aqueous uranyl acetate (45 min), dehydrated with increasing concentrations of ethanol and embedded in epoxy resin 812 (TAAB). Infiltrated samples were polymerized (2 days, 60 °C) and subsequently, the resin was detached from coverslips by alternative dipping in liquid nitrogen and hot water. Ultrathin sections (70 nm thick) were prepared in parallel to the monolayer, transferred to formvar-coated EM buttonhole grids and stained with aqueous uranyl acetate (10 min) and lead citrate (3 min).

For immunogold labeling, centrosome-enriched pellets from lambs were fixed in mild conditions (4% paraformaldehyde) and processed for embedding in Lowicryl HM23 acrylic resin. Briefly, small amounts of the chemically fixed pellets were cryoprotected with glycerol, applied to small pieces of filter paper, blotted and fast frozen by plunge-freezing in liquid ethane. Frozen specimens were transferred to a Reichert–Jung AFS freeze-substitution unit (Leica), and maintained for 60 h in pure methanol and 0.5% (w/v) uranyl acetate. The samples were then subjected to a controlled temperature increase before embedding in HM23 and polymerizing with UV light. Ultrathin (70 nm thick) sections were prepared, transferred to formvar-coated EM buttonhole grids and immunogold-labeled. After blocking with TBS/T (30 nm Tris, 150 mM NaC and 1% BSA plus 0.05% Tween), the sections were incubated with primary antibody in conditions specific to each antibody (dilution 1:5 to 1:100, for 1 to 5 h at room temperature, generally with an additional incubation overnight at 4 °C). Antibody binding was detected with 10 nm colloidal gold conjugated goat F(ab′)2 anti-mouse or anti-rabbit IgG + IgM antibodies (diluted 1:40 or 1:60, 1 to 3 h: BBInternational) and the immunolabeled sections were then counterstained with saturated uranyl acetate (10 min).

### Sample preparation for tomography

To confirm centrosome integrity and purity by negative staining, centrosomes were centrifuged on a plain carbon grid, or applied directly to plain carbon and the sucrose removed by washing with buffer. The grids were stained for 30 s with 0.1% phosphotungstic acid (PTA, pH 6.5).

For cryo-ET, centrosomes were applied to quantifoil 4/1 grids, the sucrose was washed out in several drops of buffer, the last of which included fiducial markers (10 nm BSA-coated gold beads) for tilt series alignment. The grids were manually blotted and plunge-frozen in a Leica CPC station. The grids for high quality tomography were prepared with lamb thymus centrosomes.

### Electron microscopy

The cryo grids were quality-controlled using a Tecnai G2 200 kV Field Emission Gun (FEG) microscope equipped with a CCD slowscan camera. Cryo-ET acquisition was performed on a 300 kV Titan Krios microscope (FEI) equipped with a postcolumn energy filter (968 Quantum) and a direct detection camera (K2 Summit: both from Gatan). Low-dose tilt-series acquisition (<100 e/Å2 cumulative dose) was controlled with SerialEM software^77^ using 2° tilt increments, −5 µm defocus, and 14,600 × (pixel size 3.4 Å) or 11,900 × magnification (4.21 Å). Images from negative-stained grids and ultrathin sections of resin inclusions were acquired on a Jeol JEM 1200 EXII microscope operating at 100 kV and equipped with a Gatan ES1000W CCD detector.

### Image processing

Tilt series were aligned using IMOD software^78^ and mass-normalised with PRIISM^79^. TOMOCTF was used for CTF detection and correction^80^. Tomograms were reconstructed following a SIRT algorithm with TOMO3D^81^ and for the denoising, tomograms were filtered using Situs volfltr^55^. The tomograms were Fourier filtered and background subtracted in PRIISM^79^ or NIH ImageJ^76^. A few iterations of bilateral filtering were also performed in PRIISM^79^ and 3D adaptative histogram filtering was performed in Thermo Scientific™ Amira™ ^82^.

### Sample preparation for proteomics

Only samples extracted from lamb thymus were used for the proteomics analysis. Each purified sucrose gradient fraction (40%, 50% and 70%) was centrifuged (15.000 g, 4 °C, 15 min), the pellet was resuspended and boiled in Laemmli sample buffer, and then resolved by gel electrophoresis. Entire gel lanes were excised manually from Coomassie blue-stained gels, deposited in 96-well plates and processed manually or automatically in a Proteineer DP digest robot (Bruker Daltonics). The digestion protocol was based on Shevchenko *et al*.^83^, with minor variations. Briefly, gel plugs were first washed, reduced with 10 mM DTT (dithiothreitol) in 25 mM ammonium bicarbonate solution (AmBic) and alkylated in 55 mM IAA (iodoacetamide) in a 50 mM AmBic solution. The gel pieces were rinsed and dried prior to adding trypsin (16 ng/μl in 25% ACN/50 mM ammonium bicarbonate solution: Proteomics Grade, Sigma Aldrich) and incubated at 37 °C for 6 h. The reaction was terminated by adding 0.5% TFA (trifluoroacetic acid) and the peptides were extracted, dried by speed-vacuum centrifugation and stored at −20 °C prior to LC-MS/MS analysis.

The samples were analyzed by LC-MS/MS using a nano HPLC chromatography system (Eksigent nanoLC Ultra 1D plus) coupled online to a TripleTOF 5600 mass spectrometer (SCIEX, Framingham, MA), with a nano-spray ionization source. Reverse phase chromatography was carried out using an Acclaim PepMap C18, 100 µm I.D. × 20 mm length, 5 µm particle size trapping column (ThermoFisher), and a reversed-phase, UPLC nano-Acquity column (75 µm I.D. × 150 mm length 1.7 µm particule size, 130 Å, C18: Waters). Typically, 5 µl of sample was injected and while the loading pump was operated at 2 µl/min with 0.1% formic acid (FA) in water, the nanopump provided a 250 nl/min flow-rate with linear gradient elution conditions of: 5–30% B in 60 min; 30–60% B in 10 min and 60–95% B in 1 min (A = 0.1% FA in water, B = 0.1% FA in acetonitrile ACN).

### Mass spectroscopy data acquisition

MS and MS/MS data acquisition was performed on a TripleTOF 5600 System using: ISVF = 2800 V, curtain gas = 20 psi, interface heater temperature = 150 °C, ion source gas 1 = 30 psi, declustering potential = 85 V. The data were acquired in information-dependent acquisition (IDA) mode with Analyst TF 1.6 software (AB SCIEX). For IDA parameters, 250 ms MS survey scan in the mass range of 350–1250 m/z was followed by collision-induced fragmentation (CID) of the 25 most prominent ions (MS/MS scan time = 100 ms; mass range = 100-1500 m/z). Switching criteria were (m/z) 350 < ions < m/z 1250; charge state 2–5; intensity threshold >90 counts (cps) and dynamic exclusion 20 s. Collision energy (CE) was set as rolling collision energy using a parameter script.

### Proteomic data analysis

The MS and MS/MS data from each sample fraction were processed using Peak View Software v1.1 (SCIEX) to generate peak lists in a mascot general file (mgf) format. Database searches were carried out in house using a licensed version of the Mascot Server v 2.5.0 (Matrix Science, London, UK). Searches were performed against the UniProtKB/SwissProt database (release 09.01.2015) with *Ovis aries* taxonomy restriction (UKBsp_p9940), containing 23,112 protein-coding genes and their corresponding reversed entries generated in-house using the Dbtoolkit v4.2.3 tool. Search parameters were set as follows: carbamidomethyl cysteine as fixed modification, oxidized methionines, N-terminal pyroglutamic acid and acetylation of the peptide amino termini as variable modifications. Peptide mass tolerance was set to 25 ppm in MS mode and 0.06 Da in MS/MS mode, and 2 missed cleavages were allowed. Mass accuracy was typically 10 ppm for MS and MS/MS spectra.

Searches results were exported as “.dat” files from Mascot and uploaded into the Scaffold bioinformatic tool v4.0 (Proteome Software), combining replicate analyses into the same treatment conditions, enabling sample inter-protein grouping and with False Discovery Rate (FDR) filtering using the automatic filters in the software for protein identification (FDR ≤1% at the protein level). Additional filtering criteria were: proteins identified with at least two peptides and protein identification with probability greater than 98%. Protein hits from the *Ovis Aries* database were converted to Human entries for gene enrichment analysis through the Gene Ontology Consortium tools (http://geneontology.org/) to obtain enriched biological processes. Proteins previously detected in KE37 cell centrosome samples and considered as centrosomal^58^ were also analyzed and the results compared. Otherwise, proteins obtained from lamb thymus and KE-37 cell^58^ centrosome were filtered using the information gathered about centrosomal proteins in Human Protein Atlas (http://www.proteinatlas.org, data of the cell atlas). Proteins not clearly localized to the centrosome (or MTOC) were not considered.

## Supplementary information

Supplementary Material

Suppl. Tables 1

Suppl. Tables 2

Suppl. Tables 3

Suppl. Video 1.

## Data Availability

The MS proteomic data have been deposited with the ProteomeXchange Consortium (http://www.proteomexchange.org) via the PRIDE repository^84^, with the PXD003928 dataset identifiers.

## References

[1] Bornens M (2002). Centrosome composition and microtubule anchoring mechanisms. Curr. Opin. Cell Biol..

[2] Bettencourt-Dias M, Glover DM (2007). Centrosome biogenesis and function: centrosomics brings new understanding. Nat. Rev. Mol. Cell Biol..

[3] Starr DA, Fridolfsson HN (2010). Interactions between nuclei and the cytoskeleton are mediated by SUN-KASH nuclear-envelope bridges. Annu. Rev. Cell Dev. Biol..

[4] Koonce MP, Cloney RA, Berns MW (1984). Laser irradiation of centrosomes in newt eosinophils: evidence of centriole role in motility. The Journal of Cell Biology.

[5] Ueda, M., Gräf, R., MacWilliams, H. K., Schliwa, M. & Euteneuer, U. Centrosome positioning and directionality of cell movements Proceedings of the National Academy of Sciences **94**, 9674–9678 (1997). 10.1073/pnas.94.18.9674 PMC23248 9275182

[6] Wakida NM, Botvinick EL, Lin J, Berns MW (2010). An Intact Centrosome Is Required for the Maintenance of Polarization during Directional Cell Migration. PLoS One.

[7] Bornens M (2012). The centrosome in cells and organisms. Science.

[8] Drummond IA (2012). Cilia functions in development. Curr. Opin. Cell Biol..

[9] Oh EC, Katsanis N (2012). Cilia in vertebrate development and disease. Development (Cambridge, England).

[10] Youn YH, Han Y-G (2018). Primary Cilia in Brain Development and Diseases. The American Journal of Pathology.

[11] Gönczy P (2015). Centrosomes and cancer: revisiting a long-standing relationship. Nat. Rev. Cancer.

[12] Levine MS (2017). Centrosome Amplification Is Sufficient to Promote Spontaneous Tumorigenesis in Mammals. Dev. Cell.

[13] Raff JW, Basto R (2017). Centrosome Amplification and Cancer: A Question of Sufficiency. Dev. Cell.

[14] Marthiens V, Basto R (2014). Microcephaly: STIL(l) a tale of too many centrosomes. Curr. Biol..

[15] Fu J, Hagan IM, Glover DM (2015). The centrosome and its duplication cycle. Cold Spring Harb Perspect Biol.

[16] Paintrand M, Moudjou M, Delacroix H, Bornens M (1992). Centrosome organization and centriole architecture: their sensitivity to divalent cations. J. Struct. Biol..

[17] Agircan, F. G., Schiebel, E. & Mardin, B. R. Separate to operate: control of centrosome positioning and separation. *Philos. Trans. R. Soc. Lond. B. Biol. Sci*. **369** (2014). 10.1098/rstb.2013.0461 PMC4113105 25047615

[18] Fu J, Glover DM (2012). Structured illumination of the interface between centriole and peri-centriolar material. Open Biol.

[19] Lawo S, Hasegan M, Gupta GD, Pelletier L (2012). Subdiffraction imaging of centrosomes reveals higher-order organizational features of pericentriolar material. Nat. Cell Biol..

[20] Mennella V (2012). Subdiffraction-resolution fluorescence microscopy reveals a domain of the centrosome critical for pericentriolar material organization. Nat. Cell Biol..

[21] Woodruff, J. B., Wueseke, O. & Hyman, A. A. Pericentriolar material structure and dynamics. *Philos. Trans. R. Soc. Lond. B. Biol. Sci*. **369** (2014). 10.1098/rstb.2013.0459 PMC4113103 25047613

[22] Hatch E, Stearns T (2010). The life cycle of centrioles. Cold Spring Harbor Symp. Quant. Biol..

[23] Galati D, Mitchell B, Pearson C (2016). Subdistal Appendages Stabilize the Ups and Downs of Ciliary Life. Dev. Cell.

[24] Strnad P, Gönczy P (2008). Mechanisms of procentriole formation. Trends Cell Biol..

[25] van Breugel M (2011). Structures of SAS-6 suggest its organization in centrioles. Science.

[26] Kitagawa D (2011). Structural basis of the 9-fold symmetry of centrioles. Cell.

[27] Conduit PT, Wainman A, Raff JW (2015). Centrosome function and assembly in animal cells. Nat. Rev. Mol. Cell Biol..

[28] Fry AM (1998). C-Nap1, a novel centrosomal coiled-coil protein and candidate substrate of the cell cycle-regulated protein kinase Nek2. J. Cell Biol..

[29] Bahe S, Stierhof Y-D, Wilkinson CJ, Leiss F, Nigg EA (2005). Rootletin forms centriole-associated filaments and functions in centrosome cohesion. J. Cell Biol..

[30] Tsou M-FB, Stearns T (2006). Mechanism limiting centrosome duplication to once per cell cycle. Nature.

[31] Tsou M-FB (2009). Polo kinase and separase regulate the mitotic licensing of centriole duplication in human cells. Dev. Cell.

[32] Schöckel L, Möckel M, Mayer B, Boos D, Stemmann O (2011). Cleavage of cohesin rings coordinates the separation of centrioles and chromatids. Nat. Cell Biol..

[33] Matsuo K (2012). Kendrin is a novel substrate for separase involved in the licensing of centriole duplication. Curr. Biol..

[34] Kim J, Lee K, Rhee K (2015). PLK1 regulation of PCNT cleavage ensures fidelity of centriole separation during mitotic exit. Nat. Commun..

[35] Pagan JK (2015). Degradation of Cep68 and PCNT cleavage mediate Cep215 removal from the PCM to allow centriole separation, disengagement and licensing. Nat. Cell Biol..

[36] Faragher AJ, Fry AM (2003). Nek2A kinase stimulates centrosome disjunction and is required for formation of bipolar mitotic spindles. Mol. Biol. Cell.

[37] Kim K, Lee S, Chang J, Rhee K (2008). A novel function of CEP135 as a platform protein of C-NAP1 for its centriolar localization. Exp. Cell. Res..

[38] Hardy T (2014). Multisite phosphorylation of C-Nap1 releases it from Cep135 to trigger centrosome disjunction. J. Cell Sci..

[39] He R (2013). LRRC45 Is a Centrosome Linker Component Required for Centrosome Cohesion. Cell Rep.

[40] Graser S, Stierhof Y-D, Nigg EA (2007). Cep68 and Cep215 (Cdk5rap2) are required for centrosome cohesion. J. Cell Sci..

[41] Fang G (2014). Centlein mediates an interaction between C-Nap1 and Cep68 to maintain centrosome cohesion. J. Cell Sci..

[42] Fry AM (2015). Solving the centriole disengagement puzzle. Nat. Cell Biol..

[43] Mohr L (2015). An Alternatively Spliced Bifunctional Localization Signal Reprograms Human Shugoshin 1 to Protect Centrosomal Instead of Centromeric Cohesin. Cell Rep.

[44] Cabral G, Sans SS, Cowan CR, Dammermann A (2013). Multiple mechanisms contribute to centriole separation in C. elegans. Curr. Biol..

[45] Oliveira RA, Nasmyth K (2013). Cohesin cleavage is insufficient for centriole disengagement in Drosophila. Curr. Biol..

[46] Barrera JA (2010). CDK5RAP2 regulates centriole engagement and cohesion in mice. Dev. Cell.

[47] Guichard P, Chrétien D, Marco S, Tassin A-M (2010). Procentriole assembly revealed by cryo-electron tomography. EMBO J..

[48] Guichard P (2012). Cartwheel architecture of trichonympha Basal body. Science.

[49] Li S, Fernandez J-J, Marshall WF, Agard DA (2012). Three-dimensional structure of basal body triplet revealed by electron cryo-tomography. EMBO J..

[50] Guichard P (2013). Native architecture of the centriole proximal region reveals features underlying its 9-fold radial symmetry. Curr. Biol..

[51] Greenan, G. A., Keszthelyi, B., Vale, R. D. & Agard, D. A. Insights into centriole geometry revealed by cryotomography of doublet and triplet centrioles eLife **7**, e36851 (2018). 10.7554/eLife.36851 PMC6110610 30080137

[52] Komesli S (1989). Mass isolation of calf thymus centrosomes: identification of a specific configuration. J. Cell Biol..

[53] Lange BM, Gull K (1996). A structural study of isolated mammalian centrioles using negative staining electron microscopy. J. Struct. Biol..

[54] Bai X-c, McMullan G, Scheres SHW (2015). How cryo-EM is revolutionizing structural biology. Trends Biochem. Sci..

[55] Starosolski Z, Szczepanski M, Wahle M, Rusu M, Wriggers W (2012). Developing a denoising filter for electron microscopy and tomography data in the cloud. Biophys. Rev..

[56] Irobalieva RN, Martins B, Medalia O (2016). Cellular structural biology as revealed by cryo-electron tomography. J. Cell Sci..

[57] Andersen JS (2003). Proteomic characterization of the human centrosome by protein correlation profiling. Nature.

[58] Jakobsen L (2011). Novel asymmetrically localizing components of human centrosomes identified by complementary proteomics methods. EMBO J..

[59] Bauer M, Cubizolles F, Schmidt A, Nigg EA (2016). Quantitative analysis of human centrosome architecture by targeted proteomics and fluorescence imaging. EMBO J..

[60] Bornens, M. *Structure and Functions of Isolated Centrosomesin The Centrosome*, Vitauts I. Kalnins (ed.) (Academic press, Inc, San Diego, 1992).

[61] Keller LC, Romijn EP, Zamora I, Yates JR, Marshall WF (2005). Proteomic analysis of isolated chlamydomonas centrioles reveals orthologs of ciliary-disease genes. Curr. Biol..

[62] Bettencourt-Dias M (2005). SAK/PLK4 is required for centriole duplication and flagella development. Curr. Biol..

[63] Tang C-JC (2011). The human microcephaly protein STIL interacts with CPAP and is required for procentriole formation. EMBO J..

[64] Arquint C, Nigg EA (2016). The PLK4-STIL-SAS-6 module at the core of centriole duplication. Biochem. Soc. Trans..

[65] Patzke S (2005). Identification of a novel centrosome/microtubule-associated coiled-coil protein involved in cell-cycle progression and spindle organization. Oncogene.

[66] Patzke S (2010). CSPP is a ciliary protein interacting with Nephrocystin 8 and required for cilia formation. Mol. Biol. Cell.

[67] de Saram P, Iqbal A, Murdoch JN, Wilkinson CJ (2017). BCAP is a centriolar satellite protein and inhibitor of ciliogenesis. J. Cell Sci..

[68] Uhlén M (2015). Proteomics. Tissue-based map of the human proteome. Science.

[69] Hagstrom KA, Meyer BJ (2003). Condensin and cohesin: more than chromosome compactor and glue. Nat. Rev. Genet..

[70] Sakamoto T (2011). Condensin II alleviates DNA damage and is essential for tolerance of boron overload stress in Arabidopsis. Plant Cell.

[71] Thiry, M. *Ultrastructural Detection of Nucleic Acids by Immunocytologyin Visualization of Nucleic Acids*, Gérard Morel (ed.) (CRC Press, Boca Raton, 1995).

[72] Fry AM (2002). The Nek2 protein kinase: a novel regulator of centrosome structure. Oncogene.

[73] Wade RH, Chrétien D (1993). Cryoelectron microscopy of microtubules. J. Struct. Biol..

[74] Ibrahim R, Messaoudi C, Chichon FJ, Celati C, Marco S (2009). Electron tomography study of isolated human centrioles. Microsc. Res. Tech..

[75] Chrétien D, Buendia B, Fuller SD, Karsenti E (1997). Reconstruction of the centrosome cycle from cryoelectron micrographs. J. Struct. Biol..

[76] Schneider CA, Rasband WS, Eliceiri KW (2012). NIH Image to ImageJ: 25 years of image analysis. Nat. Methods.

[77] Mastronarde DN (2005). Automated electron microscope tomography using robust prediction of specimen movements. J. Struct. Biol..

[78] Kremer JR, Mastronarde DN, McIntosh JR (1996). Computer visualization of three-dimensional image data using IMOD. J. Struct. Biol..

[79] Chen H, Hughes DD, Chan TA, Sedat JW, Agard DA (1996). IVE (Image Visualization Environment): a software platform for all three-dimensional microscopy applications. J. Struct. Biol..

[80] Fernández JJ, Li S, Crowther RA (2006). CTF determination and correction in electron cryotomography. Ultramicroscopy.

[81] Agulleiro JI, Fernandez JJ (2011). Fast tomographic reconstruction on multicore computers. Bioinformatics.

[82] Stalling, D., Westerhoff, M. & Hege, H. -C. Amira: a highly interactive system for visual data analysis (2005).

[83] Shevchenko A, Tomas H, Havlis J, Olsen JV, Mann M (2006). In-gel digestion for mass spectrometric characterization of proteins and proteomes. Nat. Protoc..

[84] Vizcaíno JA (2016). 2016 update of the PRIDE database and its related tools. Nucleic Acids Res..

