# Supplementary material for: Cryo-Electron Tomography and Proteomics studies of centrosomes from differentiated quiescent thymocytes
Source: Sci Rep. 2019 May 10;9:7187. doi: 10.1038/s41598-019-43338-9 (PMC6510768; doi:10.1038/s41598-019-43338-9)
Supplement: Supplementary file 1 — Supplementary Material [file 41598_2019_43338_MOESM1_ESM.pdf]

Cryo-Electron Tomography and Proteomics studies of centrosomes from differentiated quiescent thymocytes.

Johan Busselez[1,2*] Francisco Javier Chichón[1], Maria Josefa Rodríguez[1], Adan Alpízar[1], Séverine Isabelle Gharbi[1], Mònica Franch[1], Roberto Melero[1], Alberto Paradela[1], José L. Carrascosa[1], José-Maria Carazo[1*]

[1]Centro Nacional de Biotecnologia (CNB-CSIC), Darwin 3, Campus de Cantoblanco 28049 Madrid, Spain

[2]Current address: Institut de Génétique et de Biologie Moléculaire et Cellulaire, 1 Rue Laurent Fries, 67400 Illkirch-Graffenstaden, France

Supplementary Materials :

1) Supplementary figures (In this document) : Suppl. Fig. 1 to Suppl. Fig. 11.

2) the proteomic tables: Suppl. Tables 1.

3) the gene ontonlogy results: Suppl. Tables 2 and 3.

4) Supplementary Video 1.

Supplementary Figures Legends

Suppl. Fig. 1

a. Representatives images of immunofluorescence fields for different fractions from the second gradient: green, beta-tubulin; red, gamma-tubulin; blue, DAPI. The 40% fraction is very diluted (around 10^6-10^7 centrosomes/ml), the 50% fraction is concentrated (1-2 x10^9 centrosomes/ml), with relatively little DNA contamination, and the first 70% fractions are even more concentrated (2-3 x 10^9 centrosomes/ml) but have notable DNA contamination. For each image the upper left corner is a blow-up of the boxed region.

b. Representative negative stain EM field of the 50% fraction.

Suppl. Fig. 2

Diagram showing the quantitative distribution of the proteins detected by proteomics in the 40, 50 and 70% sucrose gradient fractions. The smaller Venn diagram is a color guide.

Suppl. Fig. 3

a. The absolute abundance of a set of centrosomal proteins was reported previously using a quantitative proteomics approach on the centrosome isolated from human KE-37 cells[1]. The graph summarizing that information (reproduction of the fig. 3 of the Bauer et al. publication[1]) is shown framed in a light blue.

The columns on left indicated the detection of those proteins in more general non quantitative proteomic studies on isolated centrosomes from in KE 37 cells[2] (Jakobsen et al. column) and herein (o.aries column)

b. A reduced set of protein involved in the inter centriole linkage as considered in the EV1 figure from Bauer et al.[1] compared in the three proteomics studies.

1. Bauer, M., Cubizolles, F., Schmidt, A. & Nigg, E. A. Quantitative analysis of human centrosome architecture by targeted proteomics and fluorescence imaging. *EMBO J.* **35**, 2152-2166 (2016).

2. Jakobsen, L. et al. Novel asymmetrically localizing components of human centrosomes identified by complementary proteomics methods. *EMBO J.* **30**, 1520-1535 (2011).

Suppl. Fig. 4

A sub-volume of inter-centriolar fibers after gentle denoising. Some slices of the sub volume are shown in which a 3D fiber network can be visualized. **a-f**, Arrowheads indicate parts of the network. Slides are ordered from **a** to **f** along the z dimension. **g** Broader view of the tomogram with localization of the sub-volume (blue box)

Suppl. Fig. 5
A sub-volume of intercentriolar fibers after gentle denoising. Some slices of the sub volume are shown in which a 3D fiber network can be visualized: black arrowheads, parts of the network; red arrowheads, amorphous density attachment points to the centriole wall. The slides are ordered from **a** to **i** along the z dimension. **j** Broader view of the tomogram with localization of the subvolume (blue box)

Suppl. Fig. 6
A subvolume of a cloud of fibers after gentle denoising. Some slides of the subvolume are shown in which a 3D fiber network can be visualized. Arrowheads indicate parts of the network. The slides are ordered from **a** to **f** along the z dimension.

Suppl. Fig. 7
A sub-volume of the distal part of a daughter centriole after gentle denoising. Some slides of the subvolume are shown in which a complex, quite irregular structure can be visualized. The slides are ordered from **a** to **f** along z dimension. **g** Broader view of the tomogram with localization of the sub-volume (blue box) .

Suppl. Fig. 8
A sub-volume of the distal part of a mother centriole after gentle denoising. Some slides of the subvolume are shown in which a complex, quite irregular structure can be visualized. The slides are ordered from **a** to **h** along the z dimension. **i** Broader view of the tomogram with localization of the subvolume (blue box).

Suppl. Fig. 9
Screening for proteins located between the proximal part of the centriole (red, beta-tubulin) with antibodies against (all green): **a** rootletin (internal fragment; H-84: Santa-Cruz), **b** rootletin (C Terminal; R145[1]), **c** LRRC45, **d** CEP215/CDK5RAP2(internal fragment; R174[2]), **e** CEP215/CDK5RAP2 (Cter: Bethyl), **f** CEP68, and **g** PCNT (all green). Merged images are shown on the right. Scale bars 1µm.

1. Bahe, S., Stierhof, Y.-D., Wilkinson, C. J., Leiss, F. & Nigg, E. A. Rootletin forms centriole-associated filaments and functions in centrosome cohesion. *J. Cell Biol.* **171**, 27-33 (2005).
2. Graser, S., Stierhof, Y.-D. & Nigg, E. A. Cep68 and Cep215 (Cdk5rap2) are required for centrosome cohesion. J. Cell Sci. 120, 4321-4331 (2007).

Suppl. Fig. 10
Immunogold labeling with antibodies against DNA:
a. antibody against dsDNA (LS-C64820/22140 LSBIO);
b. antibody against dsDNA (MA1-35346 Thermo);
c. antibody against dsDNA, ssDNA, zDNA (clone AC-30-10: CBL186 Millipore).

Suppl. Fig. 11
In silico lateral sections of centrioles. To improve the contrast, the densities have been averaged over a slice of 20 voxels extracted from a binned and denoised tomogram for each cross-section.
The green ellipse passes through the middle of the doublets/triplets and the percentage of flattening is indicated in blue. Scale bars represent 50nm.

Supplementary tables legends

Suppl. Tables 1. : Proteins identified in the three different purified centrosome fractions.

Suppl. Tables 2 and 3: Comparison of the proteomic analysis of the centrosome from KE37 cell[1] and that of centrosomes extracted from the lamb thymus as described here. This comparison was considered in term of various biological processes using gene ontology annotations, taking into account only centrosomal proteins from KE37 cells[1]. Table 2 presents the results for biological process closely associated with the centrosome, whereas Table 3 groups other processes in which the centrosome is implicated.
Human protein atlas[2] information has also been used to filter the centrosomal mapped proteins from the complete list in both studies considered (also in Table 2).

1. Jakobsen, L. et al. Novel asymmetrically localizing components of human centrosomes identified by complementary proteomics methods. *EMBO J.* **30**, 1520-1535 (2011).

2. Uhlén, M. et al. Proteomics. Tissue-based map of the human proteome. *Science* **347**, 1260419 (2015).

Supplementary video legend

Suppl. Video 1.
Z-slide by z-slide display of a high quality gently denoised cryo-tomogram of a lamb thymus centrosome. This is one of the very few cryo-tomograms where the amorphous density appears to have been stripped from the centrosome, and where the centrioles are parallel and well separated, which aids visualization of the complete inter-centriolar fiber network. The arrows point to parts of the fiber network.

Suppl. Fig. 1.

a

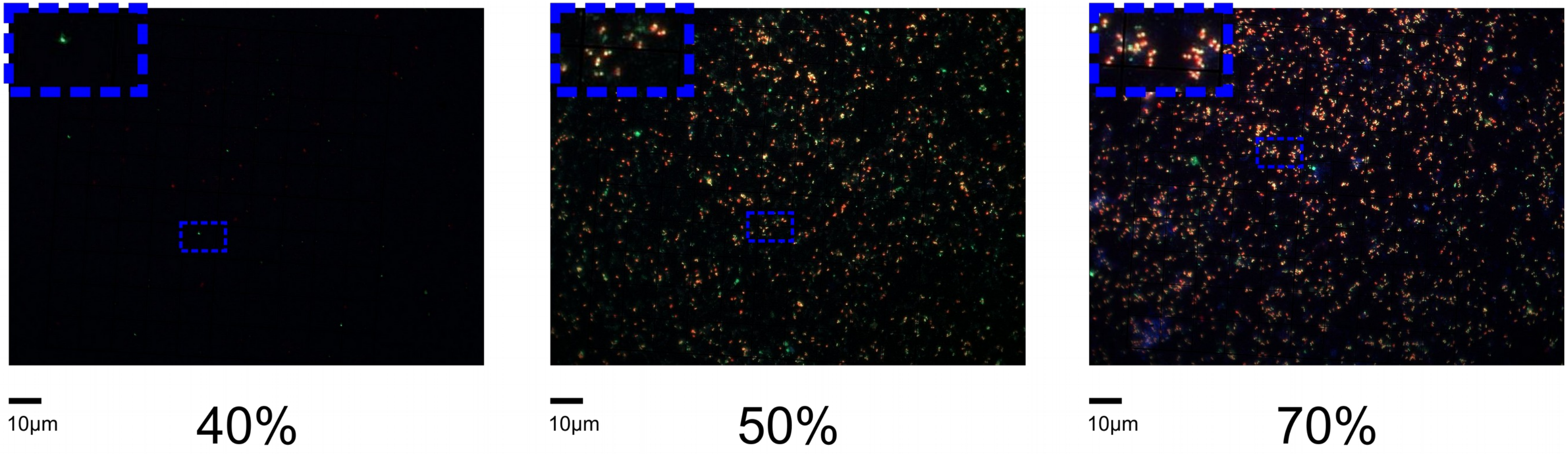

b

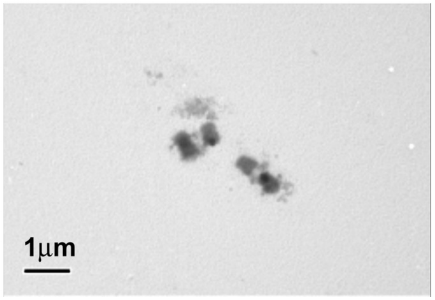

a. Representatives images of immunofluorescence fields for different fractions from the second gradient: green, beta-tubulin; red, gamma-tubulin; blue, DAPI. The 40% fraction is very diluted (around 10^6-10^7 centrosomes/ml), the 50% fraction is concentrated (1-2 x 10^9 centrosomes/ml), with relatively little DNA contamination, and the first 70% fractions are even more concentrated (2-3 x 10^9 centrosomes/ml) but have notable DNA contamination. For each image the upper left corner is a blow-up of the boxed region.

b. Representative negative stain EM field of the 50% fraction.

Suppl. Fig. 2.

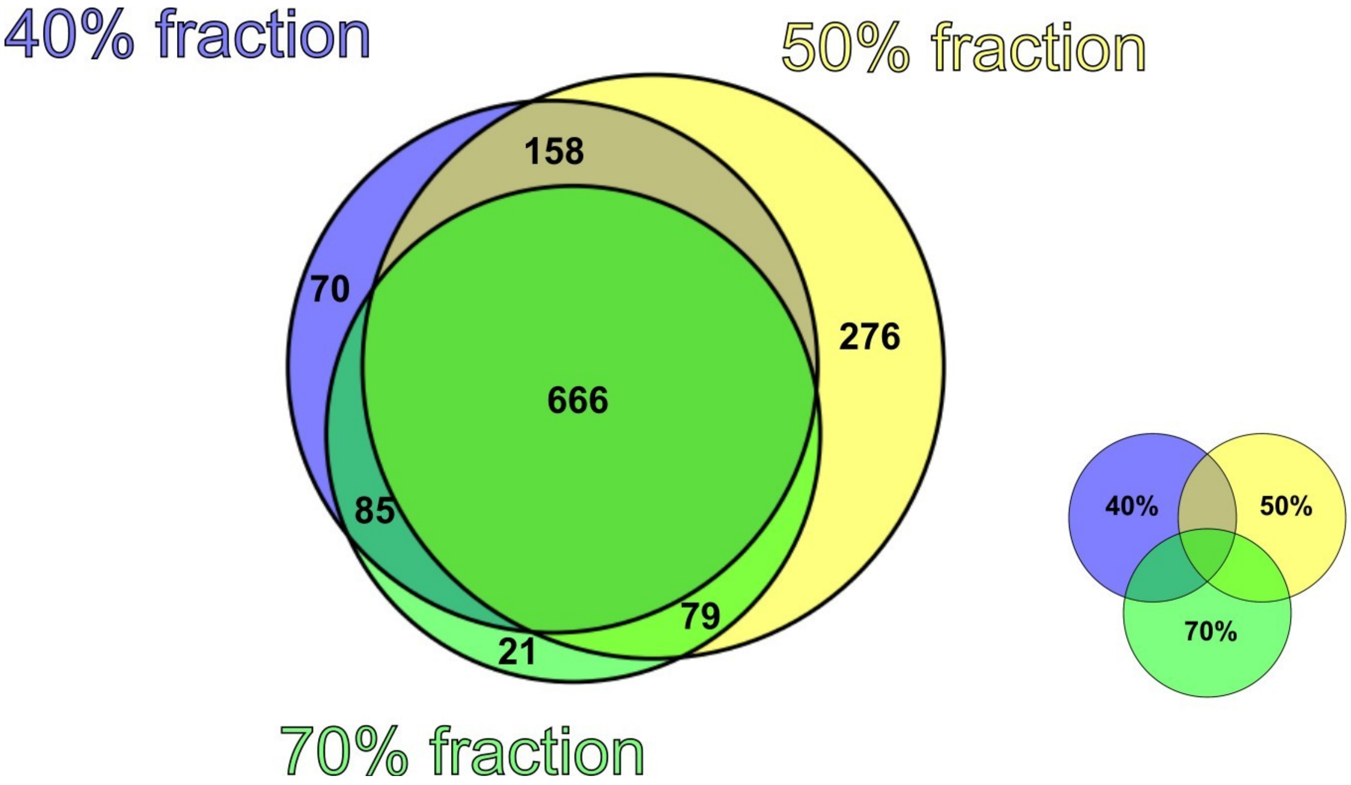

Diagram showing the quantitative distribution of the proteins detected by proteomics in the 40, 50 and 70% sucrose gradient fractions. The smaller Venn diagram is a color guide.

Suppl. Fig. 3.

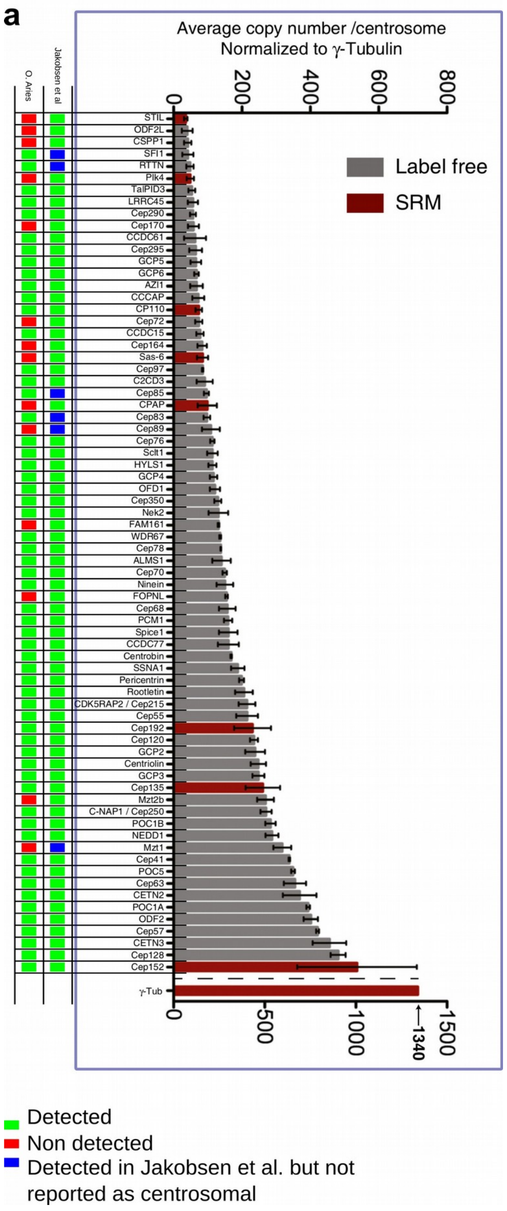

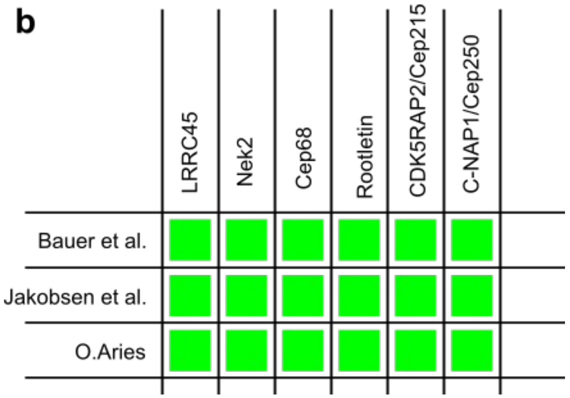

a. The absolute abundance of a set of centrosomal proteins was reported previously using a quantitative proteomics approach on the centrosome isolated from human KE-37 cells[1]. The graph summarizing that information (reproduction of the fig. 3 of the Bauer et al. publication[1]) is shown framed in a light blue.

The columns on left indicated the detection of those proteins in more general non quantitative proteomic studies on isolated centrosomes from in KE 37 cells[2] (Jakobsen et al. column) and herein (o.aries column)

b. A reduced set of protein involved in the inter centriole linkage as considered in the EV1 figure from Bauer et al.[1] compared in the three proteomics studies.

1. Bauer, M., Cubizolles, F., Schmidt, A. & Nigg, E. A. Quantitative analysis of human centrosome architecture by targeted proteomics and fluorescence imaging. *EMBO J.* **35**, 2152-2166 (2016).

2. Jakobsen, L. et al. Novel asymmetrically localizing components of human centrosomes identified by complementary proteomics methods. *EMBO J.* **30**, 1520-1535 (2011).

Suppl Fig. 4.

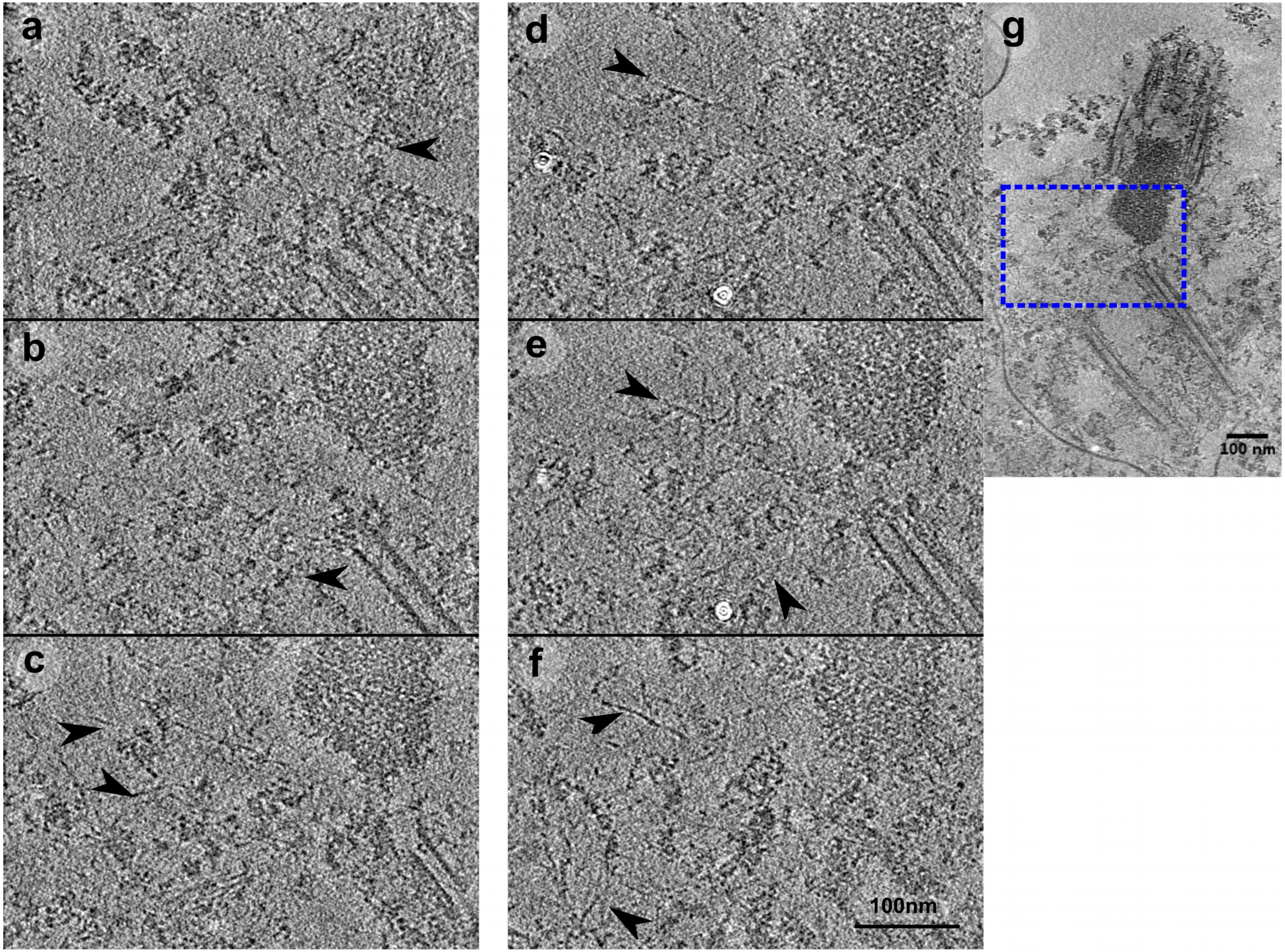

A sub-volume of inter-centriolar fibers after gentle denoising. Some slices of the sub-volume are shown in which a 3D fiber network can be visualized. **a**-**f**, Arrowheads indicate parts of the network. The slides are ordered from **a** to **f** along the z dimension. **g,** Broader view of the tomogram with localization of the sub-volume (blue box)

Suppl. Fig. 5.

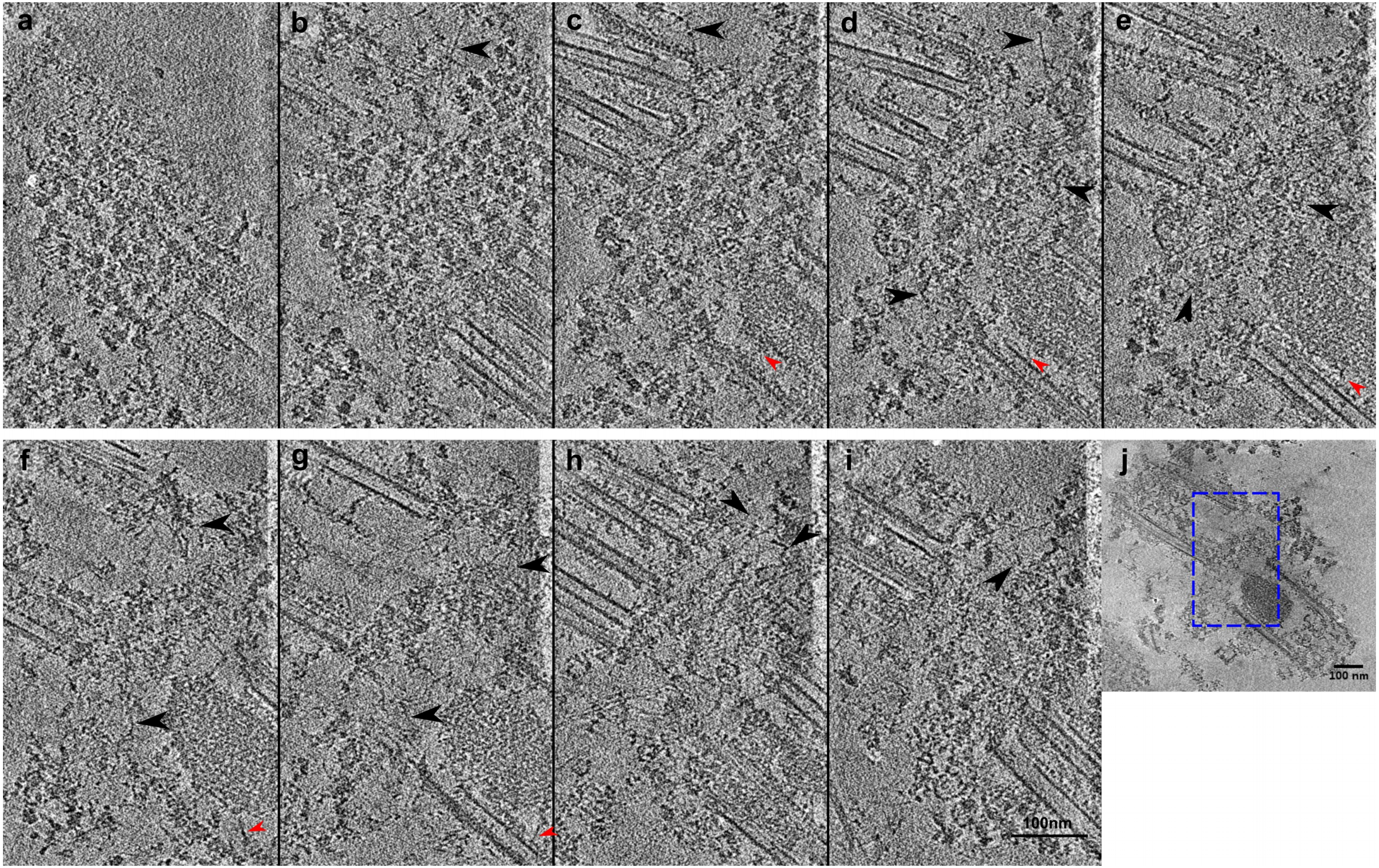

A sub-volume of inter-centriolar fibers after gentle denoising . Some slices of the sub-volume are shown in which a 3D fiber network can be visualized. Black arrowheads, parts of the network; red arrowheads, amorphous density attachment points to the centriole wall. The slides are ordered from **a** to **i** along the z dimension. **j** Broader view of the tomogram with localization of the sub-volume (blue box)

Suppl. Fig. 6.

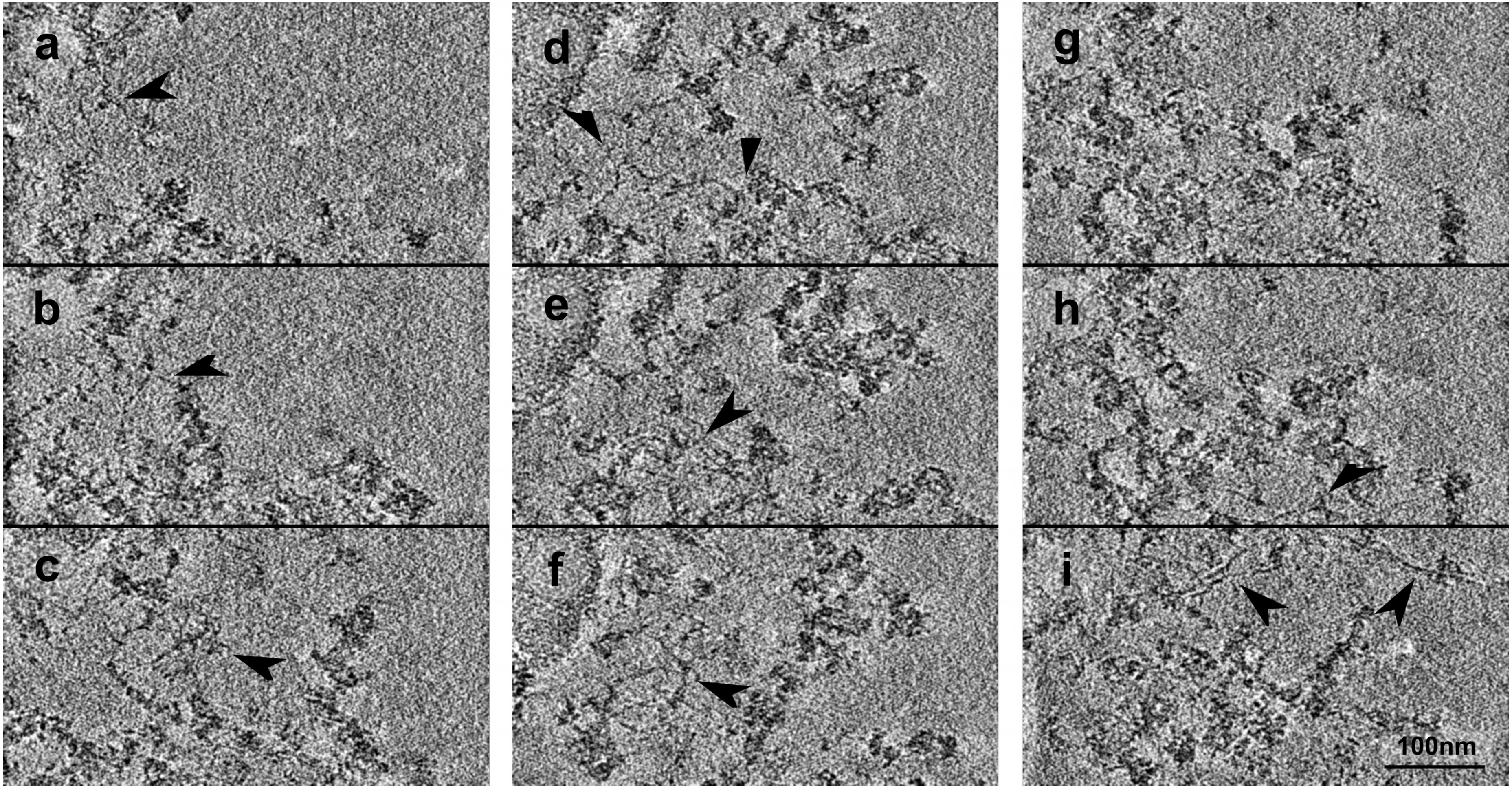

A sub-volume of cloud of fibers after gentle denoising. Some slides of the sub-volume are shown in which a 3D fiber network can be visualized. Arrowheads indicate parts of the network. The slides are ordered from **a** to **f** along the z dimension.

Suppl. Fig. 7.

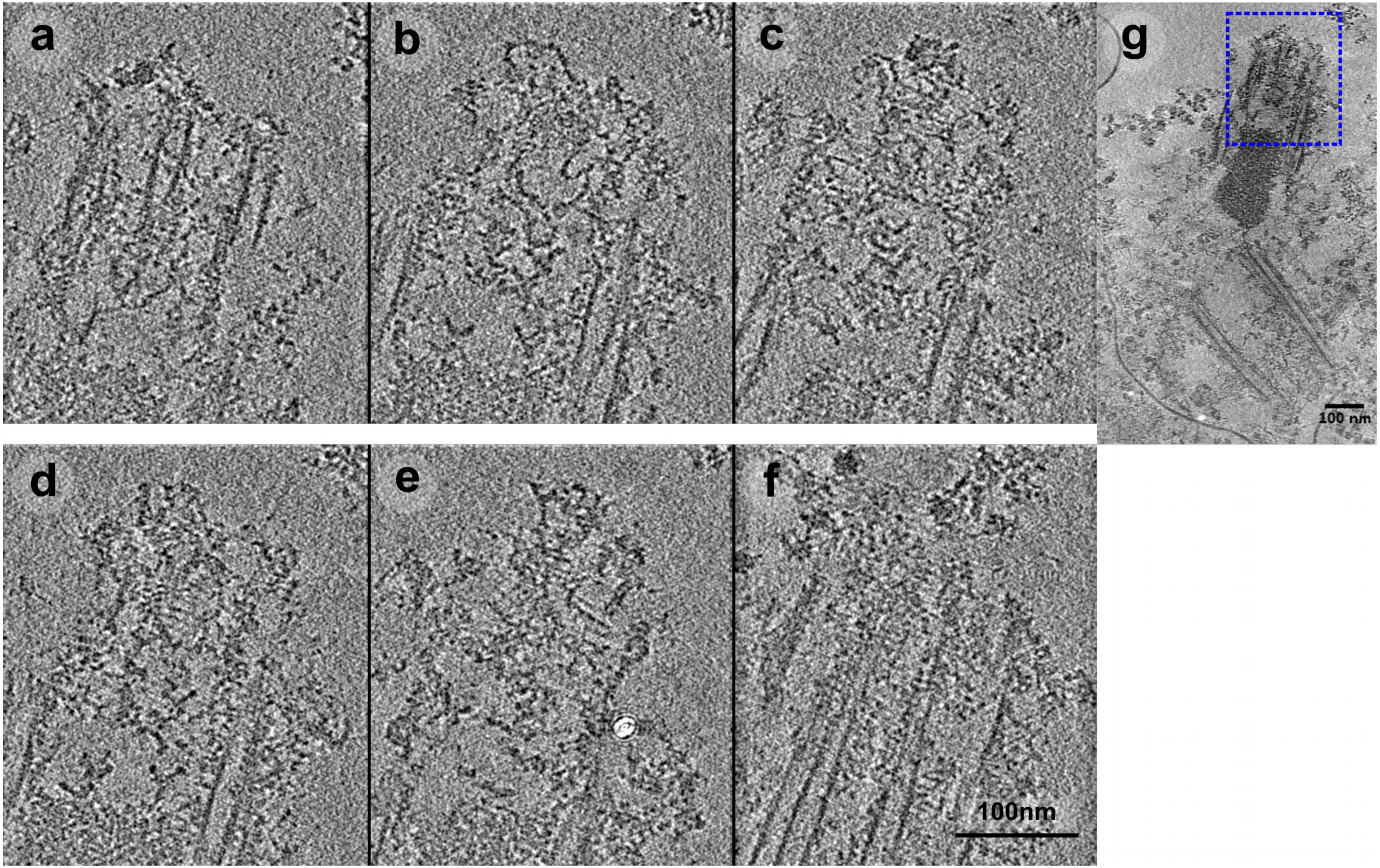

A sub-volume of the distal part of a daughter centriole after gentle denoising. Some slides of the subvolume are shown in which a complex, quite irregular structure can be visualized. The slides are ordered from **a** to **f** along the z dimension. **g** Broader view of the tomogram with localization of the sub-volume (blue box) .

Suppl. Fig. 8.

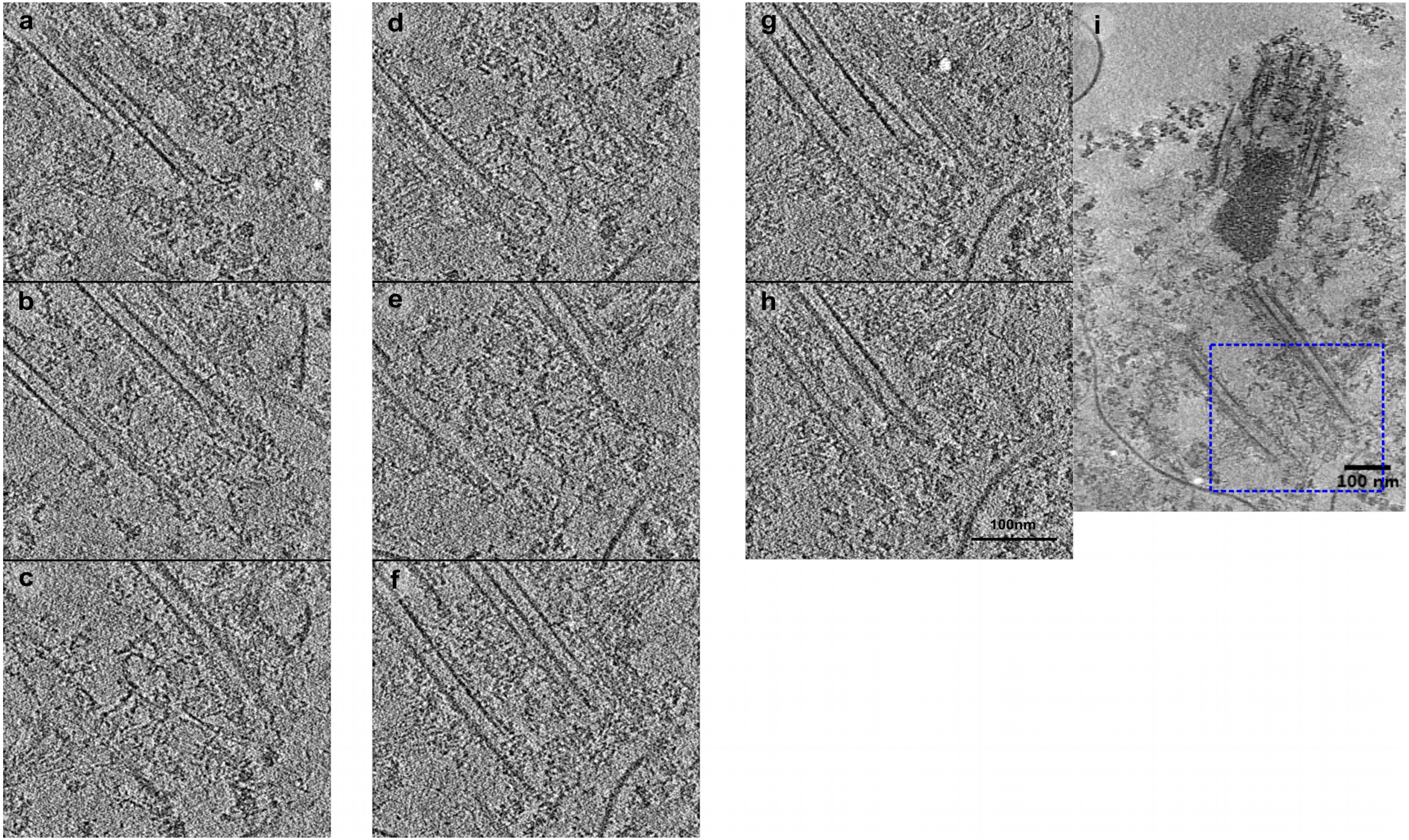

A subvolume of the a distal part of a mother centriole after gentle denoising processing. Some slides are shown of the subvolume in which a complex, quite irregular structure can be visualized. Slides ordered from **a** to **h** along z dimensions.

i broader view of the tomogram with localization of the subvolume (blue box).

Suppl. Fig. 9.

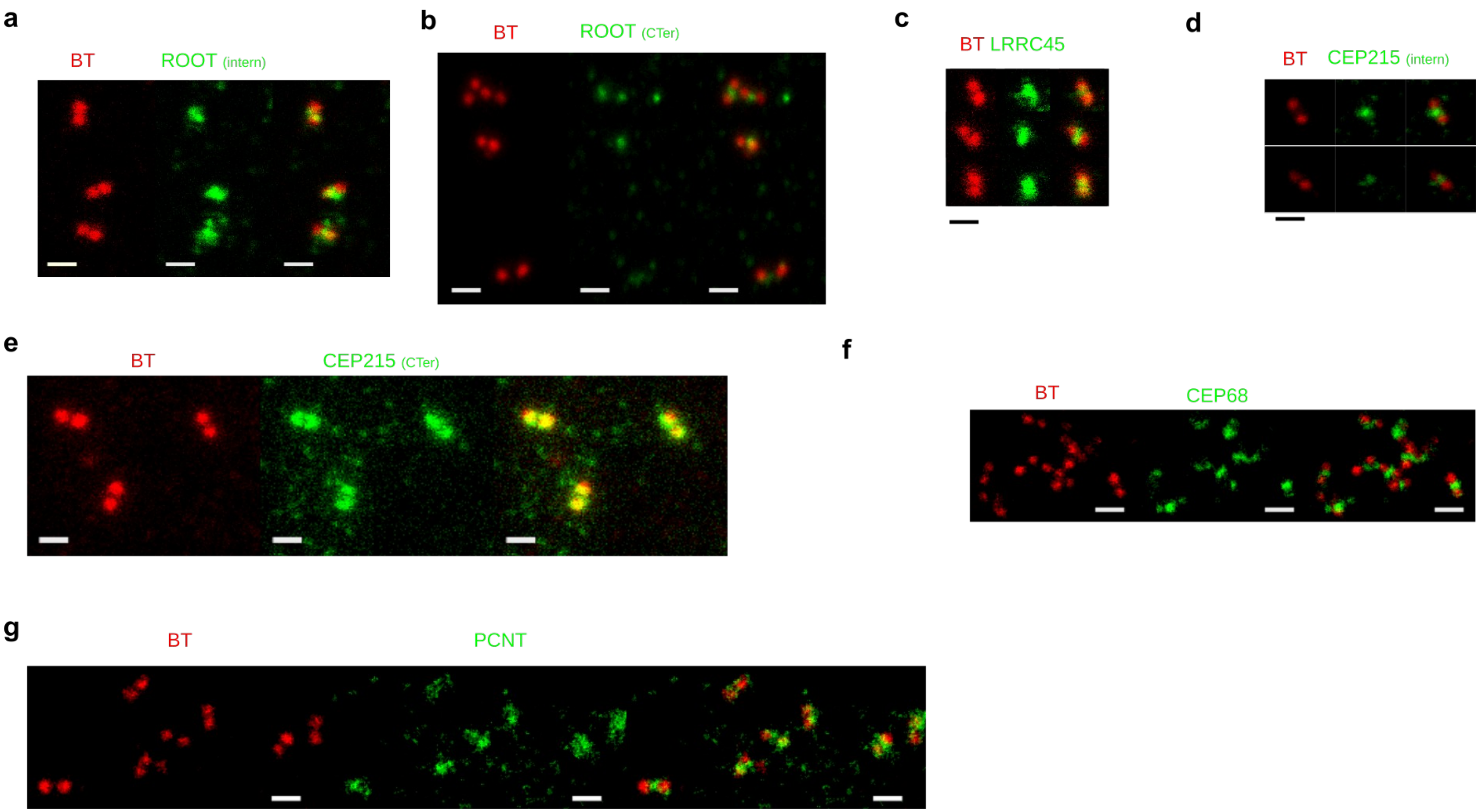

Screening for proteins located between the proximal part of the centriole (red, beta-tubulin) with antibodies against (all green): **a** rootletin (internal fragment; H-84: Santa-Cruz), **b** rootletin (C Terminal; R145[1]), **c** LRRC45, **d** CEP215/CDK5RAP2(internal fragment; R174[2]), **e** CEP215/CDK5RAP2 (Cter: Bethyl), **f** CEP68, and **g** PCNT (all green). Merged images are shown on the right. Scale bars 1 µm.

1. Bahe, S., Stierhof, Y.-D., Wilkinson, C. J., Leiss, F. & Nigg, E. A. Rootletin forms centriole-associated filaments and functions in centrosome cohesion. *J. Cell Biol.* **171**, 27-33 (2005).

2. Graser, S., Stierhof, Y.-D. & Nigg, E. A. Cep68 and Cep215 (Cdk5rap2) are required for centrosome cohesion. J. Cell Sci. 120, 4321-4331 (2007).

Suppl fig. 10.

a **b** **c**

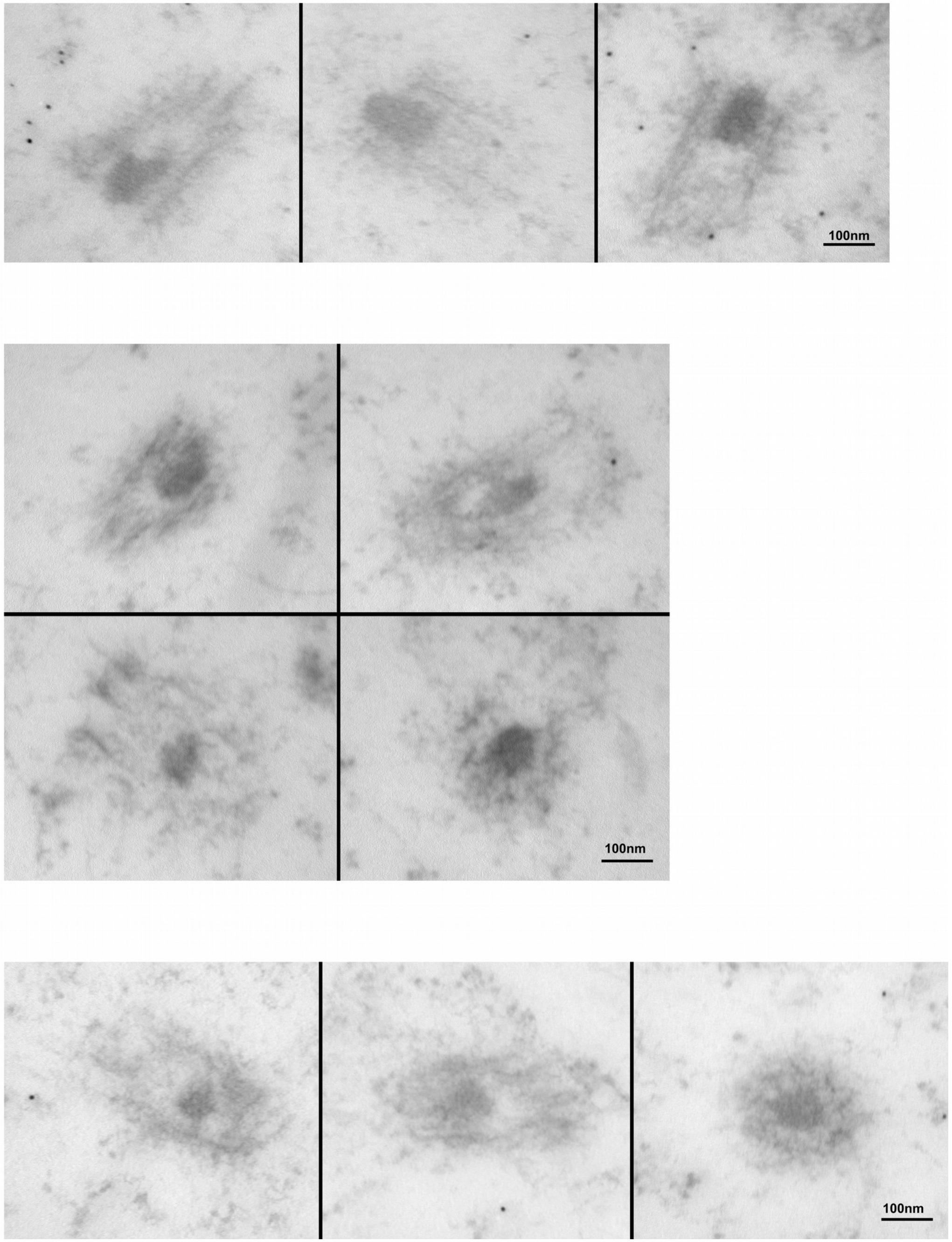

Immunogold labeling with antibody against DNA:

a. antibody against dsDNA (LS-C64820/22140 LSBIO);

b. antibody against dsDNA (MA1-35346 Thermo);

c. antibody against dsDNA, ssDNA, zDNA (clone AC-30-10: CBL186 Millipore).

Suppl fig. 11.

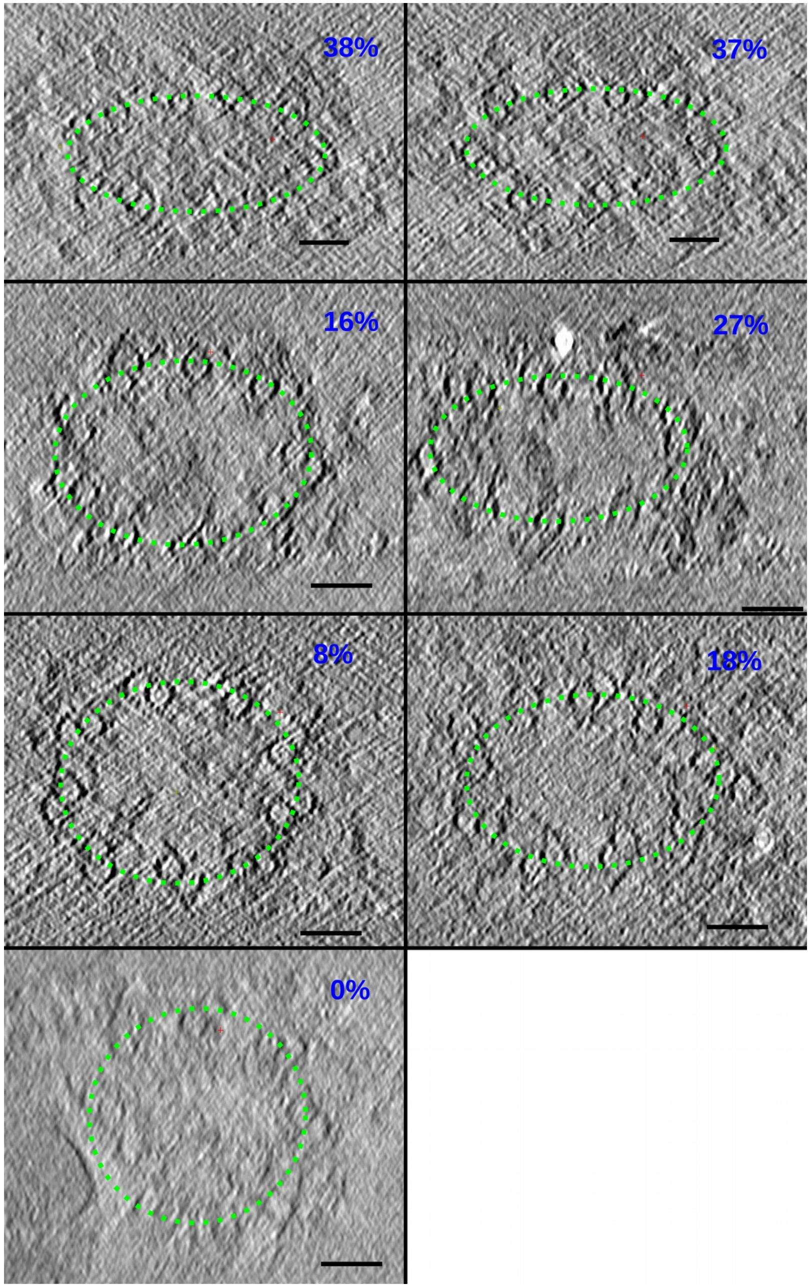

In silico lateral sections of centrioles.

To improve the contrast, the densities have been averaged over a slice of 20 voxels extracted from a binned and denoised tomogram, for each cross-section.

The green ellipse passes through the middle of the doublets/triplets and the percentage of flattening is indicated in blue. Scale bars represent 50nm.
